# Inferring gene regulatory networks from single-cell data: a mechanistic approach

**DOI:** 10.1186/s12918-017-0487-0

**Published:** 2017-11-21

**Authors:** Ulysse Herbach, Arnaud Bonnaffoux, Thibault Espinasse, Olivier Gandrillon

**Affiliations:** 1 Univ Lyon, ENS de Lyon, Univ Claude Bernard, CNRS UMR 5239, INSERM U1210, Laboratory of Biology and Modelling of the Cell, 46 allée d’Italie Site Jacques Monod, Lyon, F-69007 France; 2 Inria Team Dracula, Inria Center Grenoble Rhône-Alpes, Lyon, France; 3 Univ Lyon, Université Claude Bernard Lyon 1, CNRS UMR 5208, Institut Camille Jordan, 43 blvd. du 11 novembre 1918, Villeurbanne Cedex, F-6962 France; 4 The CoSMo company, 5 passage du Vercors, Lyon, 69007 France

**Keywords:** Single-cell transcriptomics, Gene network inference, Multiscale modelling, Piecewise-deterministic Markov processes

## Abstract

**Background:**

The recent development of single-cell transcriptomics has enabled gene expression to be measured in individual cells instead of being population-averaged. Despite this considerable precision improvement, inferring regulatory networks remains challenging because stochasticity now proves to play a fundamental role in gene expression. In particular, mRNA synthesis is now acknowledged to occur in a highly bursty manner.

**Results:**

We propose to view the inference problem as a fitting procedure for a mechanistic gene network model that is inherently stochastic and takes not only protein, but also mRNA levels into account. We first explain how to build and simulate this network model based upon the coupling of genes that are described as piecewise-deterministic Markov processes. Our model is modular and can be used to implement various biochemical hypotheses including causal interactions between genes. However, a naive fitting procedure would be intractable. By performing a relevant approximation of the stationary distribution, we derive a tractable procedure that corresponds to a statistical hidden Markov model with interpretable parameters. This approximation turns out to be extremely close to the theoretical distribution in the case of a simple toggle-switch, and we show that it can indeed fit real single-cell data. As a first step toward inference, our approach was applied to a number of simple two-gene networks simulated in silico from the mechanistic model and satisfactorily recovered the original networks.

**Conclusions:**

Our results demonstrate that functional interactions between genes can be inferred from the distribution of a mechanistic, dynamical stochastic model that is able to describe gene expression in individual cells. This approach seems promising in relation to the current explosion of single-cell expression data.

**Electronic supplementary material:**

The online version of this article (doi:10.1186/s12918-017-0487-0) contains supplementary material, which is available to authorized users.

## Background

Inferring regulatory networks from gene expression data is a longstanding question in systems biology [1], with an active community developing many possible solutions. So far, almost all studies have been based on population-averaged data, which historically used to be the only possible way to observe gene expression. Technologies now allow us to measure mRNA levels in individual cells [2–4], a revolution in terms of precision. However, the network reconstruction task paradoxically remains more challenging than ever.

The main reason is that the variability in gene expression unexpectedly stands at a large distance from a trivial, limited perturbation around the population mean. It is now clear indeed that this variability can have functional significance [5–7] and should therefore not be ignored when dealing with gene network inference. In particular, as the mean is not sufficient to account for a population of cells, a deterministic model – e.g. ordinary differential equation (ODE) systems, often used in inference [8, 9] – is unlikely to faithfully inform about an underlying gene regulatory network. Whether such a deterministic approach could still be a valid approximation or not is a difficult question that may require some biological insight into the system under consideration [10]. Another key aspect when considering individual cells is that they generally have to be killed for measurements: from a statistical point of view, temporal single-cell data therefore should not be seen as a set of time series, but rather *snapshots*, i.e. independent samples from a time series of distributions.

On the other hand, single-cell data give the opportunity of moving one step further toward a more accurate physical description of gene expression. Molecular processes of gene expression are overall now well understood, in particular transcription, but precisely how stochasticity emerges is still somewhat of a conundrum. Harnessing variability in single-cell data is expected to allow for the identification of critical parameters and also to provide hints about the basic molecular processes involved [11, 12]. Moreover, the variability arising from perturbations in cell populations is often crucial for network reconstruction to succeed [13, 14] as the deterministic inference problem suffers from intrinsic limitations [15]. From this point of view, the same information is expected to be contained in the variability between cells in single-cell data. Some of the few existing single-cell inference methods follow this path, for example using asynchronous Boolean network models [16] or generating pseudo time series [9, 17]. In this article, we use a mechanistic approach in the sense that every part of our model has an explicit physical interpretation. Importantly, mRNA observations are not used as a proxy for proteins since both are explicitly modeled.

Besides, mechanistic models that are accurate enough to describe gene expression at the single-cell level usually do not consider interactions between genes. For example, the so-called “two-state” (aka random telegraph) model has been successfully used with single-cell RNA-seq data [18], but the joint distribution of a set of genes contains much more information than the marginal kinetics of individual genes: our aim is to exploit this information while keeping the mechanistic point of view.

Namely, we propose to view the inference as a fitting procedure for a mechanistic gene network model. Whereas the goal here is not to achieve global predictability performances (e.g. as in [19]), our framework makes it possible to explicitly implement many biological hypotheses, and to test them by going back and forth between simulations and experiments. The main point of this article is to show that a tractable statistical model for network inference from single-cell data can be derived through successive relevant approximations. Finally, we demonstrate that our approach is capable of extracting enough information out of in silico-simulated noisy single-cell data to correctly infer the structures of various two-gene networks.

## Methods

In this part, we aim at deriving a tractable statistical model from a mechanistic one. We will use the two-state model for gene expression to build a “network of two-state models” by making the promoter switching rates depend on protein levels. Then, successive relevant simplifications will lead to an explicit approximation of a statistical likelihood.

### A simple mechanistic model for gene regulatory networks

#### Basic block: stochastic expression of a single gene

**Fig. 1:**
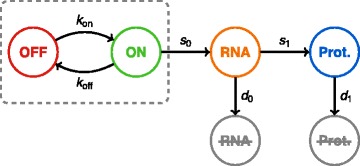
Scheme of the two-state model of gene expression. We use it as the basic block of our network model

Our starting point is the well-known two-state model of gene expression [20–23], a refinement of the model introduced by [24] from pioneering single-cell experiments [25]. In this model, a gene is described by its promoter which can be either active (on) or inactive (off) – possibly representing a transcription complex being “bound” or “unbound” but it may be more complicated [26] – with mRNA being transcribed only during the active periods. Translation is added in a standard way, each mRNA molecule producing proteins at a constant rate. The resulting model (Fig. 1) can be entirely defined by the set of chemical reactions detailed in Table 1, where chemical species *G*, *G* ^∗^, *M* and *P* respectively denote the inactive promoter, the active promoter, the amount of mRNA and proteins. The mathematical framework generally assumes stochastic mass-action kinetics [27] for all reactions, since they typically involve few molecules compared to Avogadro’s number. In this fully discrete setting, one can use the master equation to compute stationary distributions: for mRNA the exact distribution is a Beta-Poisson mixture [28], and an approximation is available for proteins when they degrade much more slowly than mRNA [29]. In addition, the time-dependent generating function of mRNA is known in closed form [30] and can be inverted in some cases to obtain the transient distribution [28].

**Table 1:** Chemical reactions defining the two-state model. The rate constants are usually abbreviated to *rates* as they correspond to actual reactions rates when only one molecule of reactant is present. In the stochastic setting, these rates are in fact propensities, i.e. probabilities per unit of time

Reaction	Rate constant	Interpretation
*G*→*G* ^∗^	*k*_on_	gene activation
*G* ^∗^→*G*	*k*_off_	gene inactivation
*G* ^∗^→*G* ^∗^+*M*	*s*_0_	transcription
*M*→*M*+*P*	*s*_1_	translation
$M \to \varnothing $	*d*_0_	mRNA degradation
$P \to \varnothing $	*d*_1_	protein degradation

In practice, the formulas involve hypergeometric series that are not straightforward to use in a statistical inference framework. Besides, these series essentially arise from the fact that such a discrete model has to enumerate all potential collisions between molecules (the stochastic mass-action assumption in the master equation). It is therefore natural to consider keeping only the most important source of noise, that is, keeping a molecular representation for rare species but describing abundant species at a higher level where molecular noise averages out to continuous quantities. A quick look at reactions in Table 1 indicates that the only rare species are *G* and *G* ^∗^, with quantities [*G*] and [*G* ^∗^] being equal to 0 or 1 molecule and satisfying the conservation relation [*G*]+[*G* ^∗^]=1. The other two, *M* and *P*, are not conserved quantities in the model and reach a much wider range in biological situations [31], meaning that saturation constants *s*_0_/*d*_0_ and *s*_1_/*d*_1_ are much larger than 1 molecule.

Hence, letting *E*(*t*), *M*(*t*) and *P*(*t*) denote the respective quantities of *G* ^∗^, *M* and *P* at time *t*, we consider a hybrid version of the previous model, where *E* has the same stochastic dynamics as before, but with *M* and *P* now following usual rate equations:

1 $$  \left\{ \begin{aligned} {E(t)} &: 0 \xrightarrow[]{k_{\text{on}}} 1, \;\; 1 \xrightarrow[]{k_{\text{off}}} 0 \\ {{M'(t)}} &= s_{0} {E(t)} - d_{0} {M(t)} \\ {{P'(t)}} &= s_{1} {M(t)} - d_{1} {P(t)} \end{aligned}\right.  $$

This system simply switches between two ordinary differential equations, depending on the value of the two-state continuous-time Markov process *E*(*t*), making it a Piecewise-Deterministic Markov Process (PDMP) [32]. From a mathematical perspective, model (1) rigorously approximates the original molecular model when *s*_0_/*d*_0_ and *s*_1_/*d*_1_ are large enough [33, 34] and interestingly, it has already been implicitly considered in the biological literature [22, 23]. Note also that the stationary distribution of mRNA is a scaled Beta distribution that is exactly the one of the Beta-Poisson mixture in the discrete model [28]. Similarly to a recent approach for a two-gene toggle switch [35], we will use (1) as a basic building block for gene networks.

When both *k*_on_≪*k*_off_ and *d*_0_≪*k*_off_, mRNA is transcribed by *bursts*, i.e. during short periods which make the mRNA quantity stay far from saturation. Hence, the amount transcribed within each burst is approximately proportional to the burst duration, whose mean is 1/*k*_off_ by definition: this justifies the quantity *s*/*k*_off_ often being called “burst size” or “burst amplitude”. Furthermore, promoter active periods are much shorter than inactive ones so they can be seen as instantaneous, justifying the name “burst frequency” for the inverse of the mean inactive time *k*_on_. We place ourselves in this situation as it often occurs in experiments [22, 23, 36–38]. Note however that these two notions are not clearly defined when relations *k*_on_≪*k*_off_ and *d*_0_≪*k*_off_ do not hold.

#### Adding interactions between genes: the network model

Now considering a given set of *n* genes, a natural way of building a network is to assume that each gene *i* produces specific mRNA *M*_*i*_ and protein *P*_*i*_, and to define a version of model (1) with its own parameters:

2 $$ \left\{ \begin{aligned} E_{i}(t) &: 0 \xrightarrow[]{ k_{\text{on},i}} 1, \;\; 1 \xrightarrow[]{ k_{\text{off},i}} 0 \\ {M_{i}}'(t) &= s_{0,i} {E_{i}(t)} - d_{0,i} {M_{i}(t)} \\ {P_{i}}'(t) &= s_{1,i} {M_{i}(t)} - d_{1,i} {P_{i}(t)} \end{aligned}\right.  $$

**Fig. 2:**
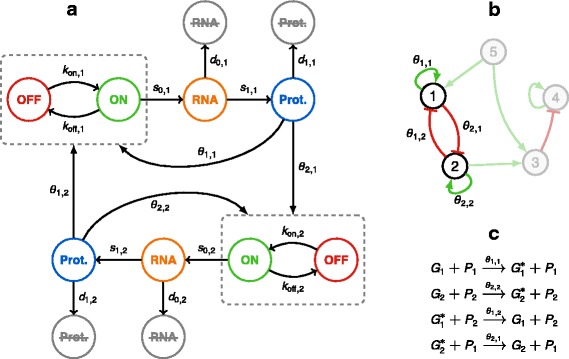
Different views of the network model. **a** Two genes interacting with each other, forming a network. Interactions are assumed to arise from the dependence of promoter dynamics on protein quantities. **b** A higher level of abstraction leads to the traditional gene regulatory network representation. **c** A toy example of reactions defining the interactions between genes 1 and 2, making the link between representations (**a**) and (**b**)

Still, genes have static parameters and do not interact with each other. To get an actual network, we need to go one step further: reactions *G*_*i*_→*G*_*i*_^∗^ and *G*_*i*_^∗^→*G*_*i*_ are not assumed to be elementary anymore, but rather represent complex reactions involving proteins so that promoter parameters *k*_on,*i*_ and *k*_off,*i*_ now depend on proteins (Fig. 2 a), and a fortiori on time. Our network model will correspond to the explicit definition, for all gene *i*, of functions *k*_on,*i*_(*P*_1_,…,*P*_*n*_) and *k*_off,*i*_(*P*_1_,…,*P*_*n*_). These functions shall also depend on network-specific parameters quantifying the interactions, thus making the link between “fitting a chemical model” and “inferring a network”. As a toy example, consider replacing *G*_*i*_→*G*_*i*_^∗^ with two parallel elementary reactions

3 $$  G_{i} \xrightarrow[]{\theta_{i,0}} {G_{i}}^{*} \quad \text{and} \quad G_{i} + P_{j} \xrightarrow[]{\theta_{i,j}} {G_{i}}^{*} + P_{j}  $$

for which applying the law of mass action directly gives *k*_on,*i*_(*P*_1_,…,*P*_*n*_)=*θ*_*i*,0_+*θ*_*i,j*_ *P*_*j*_. In a regulatory network (Fig. 2 b), it would correspond to adding a directed edge from gene *j* to gene *i*, with *θ*_*i*,0_ the basal parameter of gene *i*, and *θ*_*i,j*_ the strength of activation of gene *i* by protein *P*_*j*_. We emphasize that the action of *P*_*j*_ on the promoter *G*_*i*_ is not necessarily direct. For example, *P*_*j*_ can instead indirectly modulate the amount/activity of a transcription factor: we suppose in this article that such hidden reactions are fast enough regarding gene expression dynamics so that protein *P*_*j*_ is a relevant proxy for the transcription factor. Moreover, although we assume here that interactions can only happen at the level of *k*_on,*i*_ and *k*_off,*i*_, mainly for identifiability purposes, it is also possible to make *d*_1,*i*_ and *s*_1,*i*_ depend on proteins without fundamentally changing the mathematical approach (e.g. see [39, 40]).

In order to simplify notations, we normalize model (2) into a dimensionless equivalent model: we rewrite it in terms of new variables $\overline {M}_{i} = \frac {d_{0,i}}{s_{0,i}}M_{i}$ and $\overline {P}_{i} = \frac {d_{0,i}d_{1,i}}{s_{0,i}s_{1,i}}P_{i}$, which have values between 0 and 1, and report this scale change in the definition of *k*_on,*i*_ and *k*_off,*i*_ (see section 1.1 of Additional file 1 for details). In the remainder of this article, the new variables will still be denoted by *M*_*i*_ and *P*_*i*_ as no confusion arises. The resulting normalized model is:

4 $$  \left\{ \begin{aligned} E_{i}(t) &: 0 \xrightarrow[]{ k_{\text{on},i}} 1, \;\; 1 \xrightarrow[]{ k_{\text{off},i}} 0 \\ {M_{i}}'(t) &= d_{0,i} \left({E_{i}(t)} - {M_{i}(t)}\right) \\ {P_{i}}'(t) &= d_{1,i} \left({M_{i}(t)} - {P_{i}(t)}\right) \end{aligned}\right.  $$

still omitting the dependence of *k*_on,*i*_ and *k*_off,*i*_ on (*P*_1_(*t*),…,*P*_*n*_(*t*)) for clarity. This form enlightens the fact that *s*_0,*i*_ and *s*_1,*i*_ are just scaling constants: given a path (*E*_*i*_,*M*_*i*_,*P*_*i*_)_*i*_ of system (4), one can go back to the physical path by simply multiplying *M*_*i*_ by (*s*_0,*i*_/*d*_0,*i*_) and *P*_*i*_ by (*s*_0,*i*_/*d*_0,*i*_)×(*s*_1,*i*_/*d*_1,*i*_).

Therefore, we get a general network model where each link between two genes is directed and has an explicit biochemical interpretation in terms of molecular interactions. The previous example is very simplistic but one can use virtually any model of chromatin dynamics to derive a form for *k*_on,*i*_ and *k*_off,*i*_, involving hit-and-run reactions, sequential binding, etc. [41]. Such aspects are still far from being completely understood [42–45] and this simple network model can hopefully be used to assess biological hypotheses. In the next part, we will introduce a more sophisticated interaction form based on an underlying probabilistic model, which is both “statistics-friendly” and interpretable as a non-equilibrium steady state of chromatin environment [43].

#### Some known mathematical results

Thanks to some recent theoretical results [40, 46], simple sufficient conditions on *k*_on,*i*_ and *k*_off,*i*_ ensure that the PDMP network model (4) is actually well-defined and that the overall joint distribution of (*E*_*i*_,*M*_*i*_,*P*_*i*_)_*i*_ converges as *t*→+*∞* to a unique stationary distribution, which will be the basis of our statistical approach. Namely, we assume in this article that *k*_on,*i*_ and *k*_off,*i*_ are continuous functions of (*P*_1_,…,*P*_*n*_) and that they are greater than some positive constants. These conditions are satisfied in most interesting cases, including the above toy example (3) when *θ*_*i*,0_>0.

Contrary to creation rates *s*_0,*i*_ and *s*_1,*i*_, degradation rates *d*_0,*i*_ and *d*_1,*i*_ play a crucial role in the dynamics of the system. Intuitively, the ratios (*k*_on,*i*_+*k*_off,*i*_)/*d*_0,*i*_ and *d*_0,*i*_/*d*_1,*i*_ respectively control the buffering of promoter noise by mRNA and the buffering of mRNA noise by proteins. A common situation is when promoter and mRNA dynamics are fast compared to proteins, i.e. when *d*_0,*i*_≫*d*_1,*i*_ with (*k*_on,*i*_+*k*_off,*i*_)/*d*_0,*i*_ fixed. At the limit, the promoter-mRNA noise is fully averaged by proteins and model (4) simplifies into a deterministic system [47]:

5 $$  {P_{i}}'(t) = d_{1,i} \left(\frac{ k_{\text{on},i}(\mathbf{P}(t))}{ k_{\text{on},i}(\mathbf{P}(t)) + k_{\text{off},i}(\mathbf{P}(t))} - {P_{i}(t)}\right)  $$

where **P**(*t*)=(*P*_1_(*t*),…,*P*_*n*_(*t*)). The diffusion limit, which keeps a residual noise, can also be rigorously derived [48]. Unsurprisingly, one recovers the traditional way of modelling gene regulatory networks with Hill-type interaction functions. Equation 5 is useful to get an insight into the behaviour of the system (4) for given *k*_on,*i*_ and *k*_off,*i*_, yet it should be used with caution. Indeed, the *d*_0,*i*_/*d*_1,*i*_ ratio has been shown to span a high range, averaging out to the value *d*_0,*i*_/*d*_1,*i*_≈5 in mammalian cells [31], for which taking the limit *d*_0,*i*_≫*d*_1,*i*_ is not obvious. This is consistent with recent single-cell experiments showing a high variability of both mRNA and protein levels between cells [37]. In that sense, the PDMP model is much more robust than its deterministic/diffusion counterpart while keeping a similar level of mathematical complexity, which motivates our approach.

#### Simulation

**Fig. 3:**
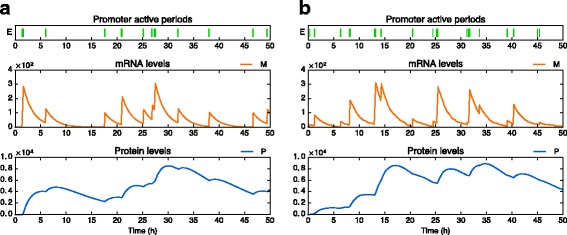
Simulations of the two-state model for a single gene. **a** Sample path of the PDMP model using our hybrid numerical scheme (computation time ≈0.05 s). **b** Sample path of the classic model using exact stochastic simulation [27] (computation time ≈10 s). Parameters values are *k*_on_=0.34, *k*_off_=10, *s*_0_=10^3^, *s*_1_=10, *d*_0_=0.5 and *d*_1_=0.1 (in h^−1^)

We propose a simple algorithm to compute sample paths of our stochastic network model (4). It consists in a hybrid version of a basic ODE solver, making it efficient enough to perform massive simulations on large scale networks involving arbitrary numbers of molecules, which would be intractable with a classic molecule-based model (Fig. 3). The deterministic part of the algorithm is a standard explicit Euler scheme, while the stochastic part is based on the transient promoter distribution for single genes: this can be justified by the fact that during a small enough time interval, proteins remain almost constant so genes behave as if *k*_on,*i*_ and *k*_off,*i*_ were constant. We therefore use Bernoulli steps, in a similar way of a diffusion being simulated using gaussian steps.

After discretizing time with step *δ* *t*, the numerical scheme is as follows. Starting from an initial state (*E*_*i*_^0^,*M*_*i*_^0^,*P*_*i*_^0^)_*i*_, the update of the system from *t* to *t*+*δ* *t* is given by:

6 $$ \left\{ \begin{aligned} {E_{i}}^{t+\delta t} &\sim \mathcal{B}\left(\pi_{i}^{t}\right) \\ {M_{i}}^{t+\delta t} &= (1 - d_{0,i}\delta t){M_{i}}^{t} + d_{0,i}\delta t {E_{i}}^{t} \\ {P_{i}}^{t+\delta t} &= (1 - d_{1,i}\delta t){P_{i}}^{t} + d_{1,i}\delta t {M_{i}}^{t} \end{aligned}\right.  $$

where the Bernoulli distribution parameter $\pi _{i}^{t}$ is derived by locally solving the master equation for the promoter [49], i.e.

$$\pi_{i}^{t} = \frac{a_{i}^{t}}{a_{i}^{t} + b_{i}^{t}} + \left({E_{i}}^{t} - \frac{a_{i}^{t}}{a_{i}^{t} + b_{i}^{t}}\right)e^{-(a_{i}^{t} + b_{i}^{t})\delta t} $$

with the notation $a_{i}^{t} = k_{\text {on},i}({P_{1}}^{t},\dots,{P_{n}}^{t})$ and $b_{i}^{t} = k_{\text {off},i}({P_{1}}^{t},\dots,{P_{n}}^{t})$. Intuitively, the algorithm is valid when *δ* *t*≪1/ max*i*{*K*_on,*i*_,*K*_off,*i*_,*d*_0,*i*_,*d*_1,*i*_} where *K*_on,*i*_ and *K*_off,*i*_ denote the maximum values of functions *k*_on,*i*_ and *k*_off,*i*_.

### Deriving a tractable statistical model

We will now adopt a statistical perspective in order to deal with gene network inference, considering a set of observed cells. If they are evolving in the same environment for a long enough time, we can reasonably assume that their mRNA and protein levels follow the stationary distribution of an underlying gene network: this distribution can be used as a statistical likelihood for the cells. Furthermore assuming no cell-cell interactions (which may of course depend on the experimental context), we obtain a standard statistical problem with independent samples. Since the stationary distribution of the stochastic network model (4) is well-defined but a priori not analytically tractable, we will derive an explicit approximation and then reduce our inference problem to a traditional likelihood-based estimation. We will do so in two cases: when there is no self-interaction, and for a specific form of auto-activation.

#### Separating mRNA and protein timescales

It is for the moment very rare to experimentally obtain the amount of proteins for many genes at the single-cell level. We will therefore assume here that only mRNAs are observed. To deal with this problem, we take the protein timescale as our reference by fixing *d*_1,*i*_ and assume that promoter dynamics are faster than proteins, i.e. (*k*_on,*i*_+*k*_off,*i*_)≫*d*_1,*i*_ in a biologically relevant way, say (*k*_on,*i*_+*k*_off,*i*_)/*d*_1,*i*_>10 (thus the deterministic limit (5) does not necessarily hold). Furthermore, in line with several recent experiments [37, 50], we assume that *d*_0,*i*_ is sufficiently larger than *d*_1,*i*_ so that the correlation between mRNAs and proteins produced by the gene is very small: model (4) then can be reduced by removing mRNA and making proteins directly depend on the promoters (see section 1.2 of Additional file 1). The result is

7 $$ \left\{ \begin{aligned} E_{i}(t) &: 0 \xrightarrow[]{ k_{\text{on},i}} 1, \;\; 1 \xrightarrow[]{ k_{\text{off},i}} 0 \\ {P_{i}}'(t) &= d_{1,i} \left({E_{i}(t)} - {P_{i}(t)}\right) \end{aligned}\right.  $$

which still admits the deterministic limit (5). Since mRNA dynamics are faster than proteins, one can also assume that, given protein levels **P**=(*P*_1_,…,*P*_*n*_), each mRNA level *M*_*i*_ follows the quasi-steady state distribution

8 $$ M_{i} \,|\, \mathbf{P} \sim \operatorname{Beta}\left(\frac{ k_{\mathrm{ on},i}(\mathbf{P})}{d_{0,i}},\frac{ k_{\text{off},i}(\mathbf{P})}{d_{0,i}}\right)  $$

corresponding to the single-gene model [28, 39] with constant parameters *k*_on,*i*_(**P**) and *k*_off,*i*_(**P**). Numerically, this approximation works well even for moderate values of *d*_0,*i*_, such as *d*_0,*i*_=5×*d*_1,*i*_ (see the “Results” section).

Biologically, Eqs. (7) and (8) suggest that correlations between mRNA levels may not directly arise from correlations between promoters *states* (which in fact are weak because of (*k*_on,*i*_+*k*_off,*i*_)≫*d*_1,*i*_), but rather originate from correlations between promoter *parameters* *k*_on,*i*_ and *k*_off,*i*_, which themselves depend on the protein joint distribution.

Table 2 sums up the successive modelling steps introduced so far. From now on, we will always assume the form (8) for the mRNA distribution, and thus our model is reduced to Eq. (7) which only involves proteins.

**Table 2:** Successive dynamical models introduced in this article. We recall for each step the main feature and the form of the mRNA stationary distribution. The full network model (step 3) is used for simulations, while the simplified one (step 4) is used to derive the approximate statistical likelihood

1	*Single-gene, discrete* [29]		
	◇ All molecules are discrete		
	◇mRNA distribution: Beta-Poisson	*↓*	Abundant species treated continuously
2	*Single-gene, PDMP* (1)		
	◇ Only the promoter is discrete		
	◇ mRNA distribution: Beta	*↓*	Introduction of interactions via *k*_on_, *k*_off_
3	*Network* (2), *normalized version* (4)		
	◇ Both accurate and fast to simulate		
	◇mRNA distribution: unknown	*↓*	Timescale separation of Protein/mRNA (*d*_0_≫*d*_1_)
4	*Simplified network* (7)		
	◇ mRNA is removed from the network		
	◇ Conditional mRNA distribution: Beta (8)		

#### Hartree approximation

In this section, we present the Hartree approximation principle and provide an explicit formula in the particular case of no self-interaction. The simplified model (7) is still not analytically tractable, but it is now appropriate for employing the *self-consistent proteomic field* approximation introduced in [51, 52] and successfully applied in [53, 54]. More precisely, we will use its natural PDMP counterpart, which will be referred to as “Hartree approximation” since the main idea is similar to the Hartree approximation in physics [51]. It consists in assuming that genes behave as if they were independent from each other, but submitted to a common “proteomic field” created by all other genes. In other words, we transform the original problem of dimension 2^*n*^ into *n* independent problems of dimension 2 that are much easier to solve (see section 2 of Additional file 1 for details).

When *k*_on,*i*_ and *k*_off,*i*_ do not depend on *P*_*i*_ (i.e. no self-interaction), this approach results in approximating the protein stationary distribution of model (7) by the function

9 $$ u(y) = \prod_{i=1}^{n} \frac{{y_{i}}^{a_{i}(y)-1} {(1-y_{i})}^{b_{i}(y)-1}}{ \operatorname{B}(a_{i}(y),b_{i}(y))}  $$

where *y*=(*y*_1_,…,*y*_*n*_)=(*P*_1_,…,*P*_*n*_)=**P**, *a*_*i*_(*y*)=*k*_on,*i*_(*y*)/*d*_1,*i*_, *b*_*i*_(*y*)=*k*_off,*i*_(*y*)/*d*_1,*i*_ and B is the standard Beta function. Note that promoter states have been integrated out since they are not required by Eq. (8).

The function *u* is a heuristic approximation of a probability density function. It is only valid when interactions are not too strong, that is, when *k*_on,*i*_ and *k*_off,*i*_ are close enough to constants, and it becomes exact when they are true constants. Besides, it does not integrate to 1 in general. However, this approximation turns out to be very robust in practice and it has the great advantage to be fully explicit (and significantly simpler than in the non-PDMP case), thus providing a promising base for a statistical model.

When *k*_on,*i*_ and *k*_off,*i*_ depend on *P*_*i*_, one can still explicitly compute the Hartree approximation in many cases: we will give an example in the next section. Alternatively, it is always possible to use formula (9) even with self-interactions, giving a correct approximation when the feedback is not too strong, as for other proteins.

#### An explicit form for interactions

We now propose an explicit definition of functions *k*_on,*i*_ and *k*_off,*i*_. Recent work [36, 55, 56] showed that apparent increased transcription actually reflects an increase in burst frequency rather than amplitude. We therefore decided to model only *k*_on,*i*_ as an actual function and to keep *k*_off,*i*_ constant. In this view, the activation frequency of a gene can be influenced by ambiant proteins, whereas the active periods have a random duration that is dictated only by an intrinsic stability constant of the transcription machinery.

Our approach uses a description of the molecular activity around the promoter in a very similar way as Coulon et al. [42]. Accordingly, we make a quasi-steady state assumption to obtain *k*_on,*i*_. This idea based on thermodynamics was also used in the DREAM3 in-Silico Challenge [57] to simulate gene networks. However, only mean transcription rate was described (instead of promoter activity in our work), which is inappropriate to model bursty mRNA dynamics at the single-cell level.

We herein derive *k*_on,*i*_ from an underlying stochastic model for chromatin dynamics. We first introduce a set of abstract chromatin states, each state being associated with one of two possible rates of promoter activation, either a low rate *k*_0,*i*_ or a high rate *k*_1,*i*_≫*k*_0,*i*_. More specifically, such chromatin states may be envisioned as a coarse-grained description of the chromatin-associated parameters that are critical for transcription of gene *i*. Second, we assume a separation of timescales between the abstract chromatin model and the promoter activity, so that the promoter activation reaction depends only on the quasi-steady state of chromatin. In other words, the effective *k*_on,*i*_ is a combination of *k*_0,*i*_ and *k*_1,*i*_ which integrates all the chromatin states: its value depends on the probability of each state and a fortiori on the transitions between them. We propose a transition scheme which leads to an explicit form for *k*_on,*i*_, based on the idea that proteins can alter chromatin by hit-and-run reactions and potentially introduce a memory component. Some proteins thereby tend to stabilize it either in a “permissive” configuration (with rate *k*_1,*i*_) or in a “non-permissive” configuration (with rate *k*_0,*i*_), providing notions of *activation* and *inhibition*. A more precise definition and details of the derivation are provided in section 3 of Additional file 1.

The final form is the following. First, we define a function of every protein but *P*_*i*_,

$$\Phi_{i}(y) = \exp(\theta_{i,i}) \prod_{j\neq i} \frac{1 + \exp(\theta_{i,j}) (y_{j}/s_{i,j})^{m_{i,j}}}{1 + (y_{j}/s_{i,j})^{m_{i,j}}} $$

which may represent the external input of gene *i*. Then, *k*_on,*i*_ is defined by

10 $$ k_{\text{on},i}(y) = \frac{k_{0,i} + k_{1,i} \Phi_{i}(y)(y_{i}/s_{i,i})^{m_{i,i}}}{1 + \Phi_{i}(y)(y_{i}/s_{i,i})^{m_{i,i}}}.  $$

**Fig. 4:**
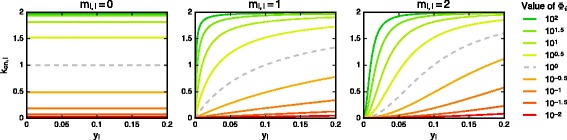
Different auto-activation types in the network model. Each color corresponds to a fixed value of *Φ*_*i*_ in formula (10), and each curve represents *k*_on,*i*_ as a function of *y*_*i*_ for *m*_*i,i*_=0 (no feedback), *m*_*i,i*_=1 (monomer-type feedback) and *m*_*i,i*_=2 (dimer-type feedback). The neutral value *Φ*_*i*_=1 is represented by a dashed gray line. Here *k*_0,*i*_=0.01, *k*_1,*i*_=2 and *s*_*i,i*_=0.1

Hence, when the input *Φ*_*i*_(*y*) is fixed, *k*_on,*i*_ is a standard Hill function which describes how gene *i* is self-activating, depending on the Hill coefficient *m*_*i,i*_ (Fig. 4). The neutral value is set to *Φ*_*i*_(*y*)=1, so that for this particular value, *s*_*i,i*_ is the usual dissociation constant. Moreover, if *θ*_*i,j*_=0 for all *j*≠*i*, then *Φ*_*i*_ becomes the constant function *Φ*_*i*_(*y*)= exp(*θ*_*i,i*_), and thus *θ*_*i,i*_ may be seen as a “basal” parameter, summing up all potential hidden inputs. On the contrary, if some *θ*_*i,j*_>0 (resp. *θ*_*i,j*_<0), then *Φ*_*i*_ becomes itself an increasing (resp. decreasing) Hill-type function of protein *P*_*j*_, where *m*_*i,j*_ and *s*_*i,j*_ again play their usual roles.

The *n*×*n* matrix *θ*=(*θ*_*i,j*_) therefore plays the same role as the interaction matrix in traditional network inference frameworks [8]. For *i*≠*j*, *θ*_*i,j*_ quantifies the regulation of gene *i* by gene *j* (activation if *θ*_*i,j*_>0, inhibition if *θ*_*i,j*_<0, no influence if *θ*_*i,j*_=0), and the diagonal term *θ*_*i,i*_ aggregates the “basal input” and the “self-activation strength” of gene *i*. Note that self-inhibition could be considered instead, but the choice has to be made before the inference since the self-interaction form is notoriously difficult to identify, especially in the stationary regime. In the remainder of this article, we assume that parameters *k*_0,*i*_, *k*_1,*i*_, *m*_*i,j*_ and *s*_*i,j*_ are known and we focus on inferring the matrix *θ*.

A benefit of the interaction form (10) is to allow for a fully explicit Hartree approximation of the protein distribution (see section 3 of Additional file 1 for details). In particular, if *m*_*i,i*_>0 and *c*_*i*_=(*k*_1,*i*_−*k*_0,*i*_)/(*d*_1,*i*_ *m*_*i,i*_) is a positive integer for all *i*, the approximation is given by

11 $$ u(y) = \prod_{i=1}^{n} \left(\sum_{r=0}^{c_{i}} p_{i,r}(y) \frac{{y_{i}}^{a_{i,r}-1} {(1-y_{i})}^{b_{i}-1}}{ \operatorname{B}(a_{i,r},b_{i})} \right)  $$

with *a*_*i,r*_=((*c*_*i*_−*r*)*k*_0,*i*_+*rk*_1,*i*_)/(*d*_1,*i*_ *c*_*i*_), *b*_*i*_=*k*_off,*i*_/*d*_1,*i*_ and

$$p_{i,r}(y) = \frac{\binom{c_{i}}{r} \operatorname{B}(a_{i,r},b_{i}) (\Phi_{i}(y)/s_{i,i}^{m_{i,i}})^{r}}{\sum_{r' = 0}^{c_{i}} \binom{c_{i}}{r'} \operatorname{B}(a_{i,r'},b_{i}) (\Phi_{i}(y)/s_{i,i}^{m_{i,i}})^{r'}}. $$

In other words, the Hartree approximation (11) is a product of gene-specific distributions which are themselves mixtures of Beta distributions: for gene *i*, the *a*_*i,r*_ correspond to “frequency modes” ranging from *k*_0,*i*_ to *k*_1,*i*_, weighted by the probabilities *p*_*i,r*_(*y*). It is straightforward to check that inhibitors tend to select the low burst frequencies of their target (*a*_*i,r*_≈*k*_0,*i*_) while activators select the high frequencies (*a*_*i,r*_≈*k*_1,*i*_). If *m*_*i,i*_=0 for some *i*, then *k*_on,*i*_ does not depend on *P*_*i*_ so one just has to replace the *i*-th term in the product (11) with the single Beta form as in Eq. (9), which is equivalent to taking the limit *c*_*i*_→+*∞*. Finally, when *m*_*i,i*_>0 but *c*_*i*_ is not an integer, using ⌈*c*_*i*_⌉ instead keeps a satisfying accuracy.

#### The statistical model in practice

Our statistical framework simply consists in combining the timescale separation (8) and the Hartree approximation (11) into a standard hidden Markov model. Indeed, conditionally to the proteins, mRNAs are independent and follow well-defined Beta distributions

12 $$ v(x,y) = \prod_{i=1}^{n} \frac{{x_{i}}^{\widetilde{a}_{i}(y)-1} {(1-x_{i})}^{\widetilde{b}_{i}(y)-1}}{ \operatorname{B}(\widetilde{a}_{i}(y),\widetilde{b}_{i}(y))}  $$

where *x*=(*x*_1_,…,*x*_*n*_)=(*M*_1_,…,*M*_*n*_)=**M**, $\widetilde {a}_{i}(y) = { k_{\mathrm { on},i}(y)}/{d_{0,i}}$ and $\widetilde {b}_{i}(y) = { k_{\text {off},i}(y)}/{d_{0,i}}$. Then one can use (11) to approximate the joint distribution of proteins. Hence, recalling the unknown interaction matrix *θ*, the inference problem for *m* cells with respective levels (**M**_*k*_,**P**_*k*_)_1≤*k*≤*m*_ is based on the (approximate) complete log-likelihood:

13 $$ \begin{aligned} \ell &= \ell(\mathbf{M}_{1},\dots,\mathbf{M}_{m}, \mathbf{P}_{1},\dots,\mathbf{P}_{m} | \theta) \\ & = \sum_{k=1}^{m} \log(u(\mathbf{P}_{k})) + \log(v(\mathbf{M}_{k},\mathbf{P}_{k})) \end{aligned}  $$

where we used conditional factorization and independence of the cells.

The basic statistical inference problem would be to maximize the marginal likelihood of mRNA with respect to *θ*. Since this likelihood has no simple form, a typical way to perform inference is to use an Expectation-Maximization (EM) algorithm on the complete likelihood (13). However, the algorithm may be slow in practice because of the computation of expectations over proteins. A faster procedure consists in simplifying these expectations using the distribution modes: the resulting algorithm is often called “hard EM” or “classification EM” and is used in the “Results” section. Moreover, it is possible to encode some potential knowledge or constraints on the network by introducing a prior distribution *w*(*θ*). In this case, from Baye’s rule, one can perform *maximum a posteriori* (MAP) estimation of *θ* by using the same EM algorithm but adding the penalization term log(*w*(*θ*)) to *ℓ* during the Maximization step (see section 4 of Additional file 1 and the “Results” section). Alternatively, a full bayesian approach, i.e. sampling from the posterior distribution of *θ* conditionally to (**M**_1_,…,**M**_*m*_), may also be considered using standard MCMC methods.

Taking advantage of the latent structure of proteins, we can also deal with missing data in a natural way: if the mRNA measurement of gene *i* is invalid in a cell *k* owing to technical problems, it is possible to ignore it by removing the *i*-th term in the conditional distribution of mRNAs (12). This only modifies the definition of *v* for cell *k* in Eq. (13), ensuring that all valid data is effectively used for each cell.

## Results

In this part, we first compare the distribution of the mechanistic model (4) to the mRNA quasi-steady state combined with Hartree approximation for proteins, on a simple toggle-switch example. Then, we show that the single-gene model with auto-activation can fit marginal mRNA distributions from real data better than the constant- *k*_on_ model. Finally, we successfully apply the inference procedure to various two-gene networks simulated from the mechanistic model.

### Relevance of the approximate likelihood

**Fig. 5:**
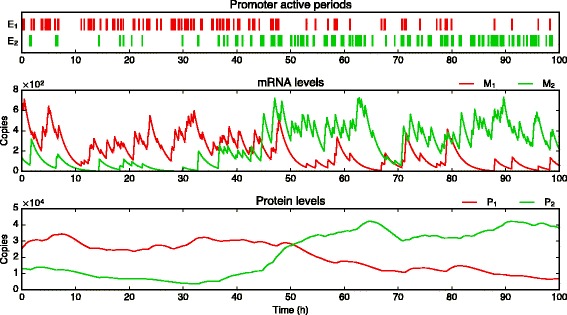
Sample path of a two-gene toggle switch. The first gene is plotted in red and the second in green. While always staying in a bursty regime regarding mRNAs, genes can switch between high and low frequency modes (here at *t*≈50 h). From this example, it is clear that the overall joint distribution can contain correlations even if the bursts themselves are not coordinated

**Fig. 6:**
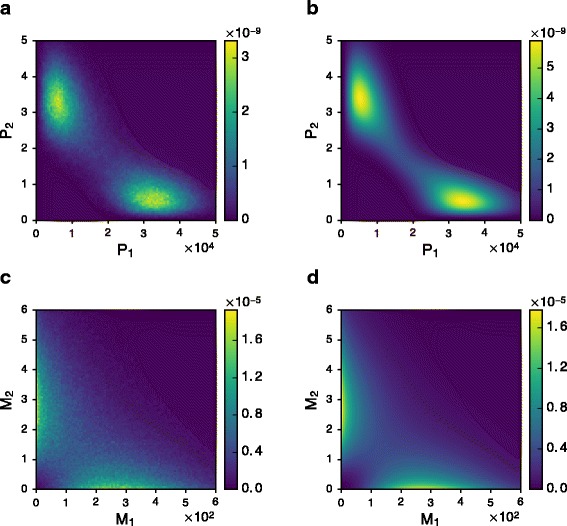
Exact and approximate stationary distributions for the example of toggle switch. True distributions (left side) were estimated by sample path simulation, while approximations (right side) have explicit formulas. **a** True distribution of proteins. **b** Approximate distribution of proteins, from formula (11). **c** True distribution of mRNAs. **d** Approximate distribution of mRNAs, obtained by integrating the conditional distribution of mRNA (12) against (**b**)

Starting from the normalized mechanistic model (4), two approximations were used to derive the final statistical likelihood (13): the quasi-steady state assumption for mRNAs given protein levels, and the Hartree approximation for the joint distribution of proteins. Crucially, this approximate likelihood has to be close enough to the exact one in order to preserve the equivalence between inferring a network and fitting the mechanistic model. To get an idea of the accuracy, we considered a basic two-gene toggle switch defined by *k*_on,*i*_ following Eq. (10) with the interaction matrix given by *θ*_1,1_=*θ*_2,2_=4 and *θ*_1,2_=*θ*_2,1_=−8 (full parameter list in section 6 of Additional file 1). By computing sample paths (Fig. 5), we estimated the stationary distribution and compared it with our approximation, which appeared to be very satisfying, both for proteins and mRNAs (Fig. 6).

### Fitting marginal mRNA distributions from real data

A particularity of single-cell data is to often exhibit bursty regimes for mRNA (meaning *k*_on_≪*k*_off_ and *d*_0_≪*k*_off_) and potentially also for proteins (adding *d*_1_≪*k*_off_), which are well fitted by Gamma distributions [37]. At this stage, it is worth mentioning that the Gamma distribution can be seen as a limit case of the Beta distribution. Intuitively, when *b*≫1 and *b*≫*a* (typically *a*=*k*_on_/*d*_0_ and *b*=*k*_off_/*d*_0_), most of the mass of the distribution Beta(*a,b*) is located at *x*≪1 so we have the first order approximation

$$\begin{array}{*{20}l} x^{a-1}(1-x)^{b-1} &= x^{a-1}\exp((b-1)\log(1-x)) \\ & \approx x^{a-1}\exp(-bx) \end{array} $$

and thus Beta(*a,b*)≈*γ*(*a,b*). This way, formulas (11) and (12) can be easily transformed into Gamma-based distributions. Parameters *s*_0_ and *k*_off_ then aggregate in *k*_off_/*s*_0_ because of the scaling property of the Gamma distribution, so only this ratio has to be inferred: from an applied perspective, it simply represents a scale parameter for each gene. This remark leads to a possible preprocessing phase that can be used for estimating the crucial basal parameters of the network, without requiring the knowledge of such scale parameters (see section 5 of Additional file 1).

**Fig. 7:**
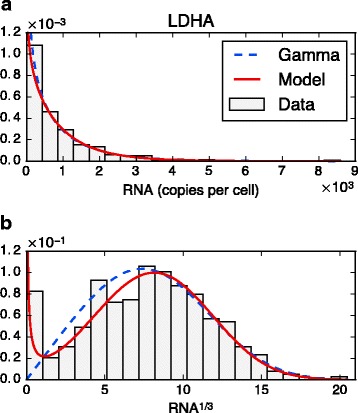
Fitting marginal distributions from real single-cell data: example of the LDHA gene. The red curve is the stationary distribution associated with our interaction form (here a single gene with auto-activation), while the dashed blue curve corresponds to the basic two-state model in the bursty regime (Gamma distribution). **a** The raw data seems to be well fitted by the Gamma distribution, which in this view is close to our model. **b** Same fit viewed after applying the transformation *x*↦*x* ^1/3^. The data becomes bimodal and the fit appears to be better with the auto-activation model

In addition, our network model is able to generate multiple modes while keeping such bursty regimes (Fig. 5), as noticeable in the stationary distribution (11). Interestingly, this feature has already been considered in the literature by empirically introducing mixture distributions [58, 59]. As a first step toward applications, we compared our model in the simplest case (independent genes with auto-activation) to marginal distributions of single-cell mRNA measurements from [38]. Our model was fitted and compared to the basic two-state model in the bursty regime, i.e. to a simple Gamma distribution: Fig. 7 shows the example of the LDHA gene. Although very close when viewed in raw molecule numbers, the distributions differ after applying the transformation *x*↦*x* ^*α*^ with *α*=1/3, which tends to compress great values while preserving small values. The data becomes bimodal, suggesting the presence of two bursting regimes, a “normal” one and a very small “inhibited” one: the auto-activation model then performs better than the simple Gamma, which necessarily stays unimodal for 0<*α*<1. Note that the RTqPCR protocol used in [38] was shown to be far more sensitive than single-cell RNA-seq in the detection of low abundance transcripts [60]. Since the data also contains small nonzero values, this tends to support a true biological origin for the peak in zero. Besides, the case of distributions that are not bimodal until transformed also arises for proteins [61].

### Application of the inference procedure

By construction of the mechanistic model, the interaction matrix *θ* can describe any oriented graph by explicitly defining causal quantitative links between genes, which is difficult to do within traditional statistical frameworks (e.g. bayesian networks or undirected Markov random fields). The logical downside is that identifiability issues seem inevitable. In a first attempt to assess this aspect, we implemented the inference method presented above and tested it on various two-gene networks, assuming auto-activation for each gene (i.e. *m*_*i,i*_>0) with Eq. (10) to maximize variability without considering perturbations of the system (parameter list in section 6 of Additional file 1).

We decided to investigate the worst case scenario in terms of cell numbers. We are fully aware of the existence of technologies allowing to interrogate thousands of cells simultaneously, but most of the recent studies still rely upon a much smaller number of cells. For each network, we therefore simulated mRNA snapshot data for 100 cells using the full PDMP model (4). We then inferred the matrix *θ* using a “hard EM” algorithm based on the likelihood (13), that is, alternatively maximizing the likelihood with respect to *θ* and with respect to the (unknown) protein levels of each cell. A lasso-like penalization term, corresponding to a prior distribution, was added to the *θ*_*i,j*_ for *i*≠*j* to obtain true zeros – so that the inferred network topology is clear – and to prevent keeping both *θ*_*i,j*_ and *θ*_*j,i*_ when one is significantly weaker (see section 4 of Additional file 1 for details of the penalization and the whole procedure).

**Fig. 8:**
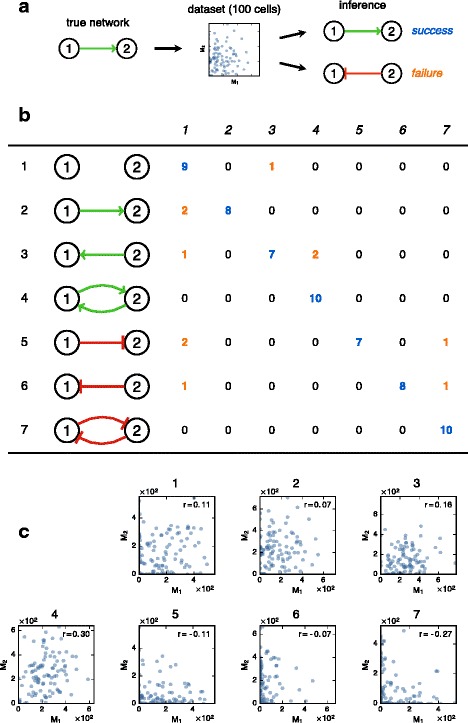
Testing our inference method on simple networks. **a** For each network, numbered from 1 to 7, we simulated 100 cells using the full mechanistic model until the stationary regime was reached. Then we took a snapshot of their mRNA levels and inferred the parameters from this data. The result was called successful when the inferred structure (topology and nature of the links) was the same as the true network. **b** For each network (rows), 10 datasets were simulated and the results were reported by counting the number of inferred *θ* corresponding to each structure (columns), highlighting successes (blue) and failures (orange). The perfect inference would lead to 10 for all the diagonal terms and 0 everywhere else. **c** Examples of simulated mRNA datasets (one for each network). Although having coherent signs, Pearson’s correlation coefficients (top right of each plot) would clearly be insufficient to distinguish between the different networks

We obtained highly encouraging results since every structure was inferred with a high probability of success (Fig. 8), meaning that the non-diagonal (i.e. interaction) terms of *θ* had the right sign and were nonzero at the right places. A list of the inferred values is provided in Additional file 1: Table S3. It is very important at that stage to emphasize that we are not trying to infer *θ* exactly: we only assess whether it has a zero or nonzero value and its sign. Although the results tend to support the identifiability of the full matrix *θ* in this simple two-gene case, one has to be aware that the quantity we maximize (an approximate likelihood) is a priori non convex and can have several local maxima (i.e. networks that are relevant candidates to explain the data). The result of the inference thus can depend on the starting point: in this first approach we chose the null matrix to be the starting point for *θ*, which corresponds to the – biologically relevant – expectation of “balanced” behaviors (e.g. we do not expect *θ*_1,1_≪*θ*_2,2_). Alternatively, one can consider some probabilistic prior knowledge on *θ* to implement a (possibly rough) idea of parameter values from a Bayesian viewpoint: it is worth mentioning that any knockout information can be implemented this way in our model.

Finally, we assessed the inference behavior in the presence of dropouts, i.e. genes expressed at a low level in a cell that give rise to zeros after measurement [4]. Our first tests tend to indicate that our approach is robust regarding dropouts, in the sense that up to 30% of simulated dropouts does not drastically affect the estimation of *θ* once the other parameters have been estimated correctly (see Additional file 1: Table S4 for an example).

## Discussion

In this paper, we introduce a general stochastic model for gene regulatory networks, which can describe bursty gene expression as observed in individual cells. Instead of using ordinary differential equations, for which cells would structurally all behave the same way, we adopt a more detailed point of view including stochasticity as a fundamental component through the two-state promoter model. This model is but a simplification of the complexity of the real molecular processes [42]. Modifications have been proposed, from the existence of a refractory period [23] to its attenuation by nuclear buffering [62]. In bacteria, the two states originate from the accumulation of positive supercoiling on DNA which stops transcription [63]. In eukaryotes, although its molecular basis is not quite understood, the two-state model is a remarkable compromise between simplicity and the ability to capture real-life data [18, 22, 36–38]. Our PDMP framework appears to be conceptually very similar to the *random dynamical system* proposed in [64] but it has two major advantages: time does not have to be discretized, and the mathematical analysis is significantly easier. We also note that a similar framework appears in [65, 66] and that a closely related PDMP – which can be seen as the limit of our model for infinitely short bursts – has recently been described in [67].

We then derive an explicit approximation of the stationary distribution and propose to use it as a statistical likelihood to infer networks from single-cell data. The main ingredient is the separation of three physical timescales – chromatin, promoter/RNA, and proteins – and the core idea is to use the self consistent proteomic field approximation from [51, 52] in a slightly simpler mathematical framework, providing fully explicit formulas that make possible the massive computations usually needed for parameter inference. From this viewpoint, it is a rather simple approach and we hope it can be adapted or improved in more specific contexts, for example in the study of lineage commitment [68]. Besides, the main framework does not necessarily has to include an underlying chromatin model and thus it can in principle also be used to describe gene networks in procaryotes.

### Mechanistic modelling and statistical inference

An important quality of the PDMP network model is that the simulation algorithm is comparable in speed with classic ODE and diffusion systems, while providing an effective approximation of the “perfect”, fully discrete, molecular counterpart [33, 35]. It is worth noticing that the PDMP – at least the promoter-mRNA system – naturally appears as an example of Poisson representation [28, 69], that is, not a simple approximation but rather the core component of the *exact* distribution of the discrete molecular model. Furthermore, such a simulation speed allowed us to compare our approximate likelihood with the true likelihood for a simple two-gene toggle switch, giving excellent results (Fig. 6). This obviously does not constitute a proof of robustness for every network: a proper quantitative (theoretical or numeric) comparison is beyond the scope of this article but would be extremely valuable. Intuitively, it should work for any number of genes, provided that interactions are not too strong.

Besides, some widely used ODE frameworks [8, 17, 57] can be seen as the fast-promoter limit of the PDMP model: this limit may not always hold in practice, especially in the bursty regime. In particular, Fig. 5 highlights the risk of using mRNA levels as a proxy for protein levels. It also explains why ordering single-cell mRNA measurements by pseudo-time may not always be relevant, as found in [38]. In [70], the authors use a hybrid model of gene expression to infer regulatory networks: it is very close to the diffusion limit of our reduced model (7) with the difference that the discrete component, called “promoter” by the authors, would correspond to the “frequency mode” in the present article, as visible for proteins in Fig. 5. From such a perspective, our approach adds a description of bursty mRNA dynamics that allows for fitting single-cell data such as in Fig. 7.

Finally, our method performed well for simple two-gene networks (Fig. 8), showing that part of the causal information remains present in the stationary distribution: this suggests that it is indeed possible to retrieve network structures with a mechanistic interpretation, even from bursty mRNA data.

### Perspectives

We focused here on presenting the key ideas behind the general network model and the inference method: the logical next step is to apply it to real data and with a larger number of genes, which is the subject of work in progress in our group. In particular, we propose a functional preprocessing phase, detailed in section 5 of Additional file 1, that only requires the knowledge of the ratio *d*_0,*i*_/*d*_1,*i*_ to estimate all the relevant parameters before inferring *θ*. The ratio between protein and mRNA degradation rates (or half-lives) hence appears to be the minimum required for such a mechanistic approach to be relevant. Depending upon the species, mRNA and protein half-lives values can be found in the literature (see e.g. [31] for human proteins half-lives), or should be estimated from ad hoc experiments.

From a computational point of view, the main challenge is the algorithmic complexity induced by the fact that proteins are not observed and have to be treated as latent variables. There is a priori no possibility of reducing this without loosing too much accuracy, and therefore some finely optimized algorithms may be required to make the method scalable. Furthermore, the identifiability properties of the interaction matrix *θ* seem difficult to derive theoretically. In this paper we focused on the stationary distribution for simplicity: importantly, several aspects such as time dependence (computing the Hartree approximation in transitory regime) or perturbations (changing the cell’s medium or performing knockouts [71], which can be naturally embedded in our framework) could greatly improve the practical identifiability.

From a biological point of view, our model does not really describe individual cells but rather a concatenation of trajectories obtained by following cells throughout divisions. Experiments suggest that it should be a relevant approximation, providing one considers mRNA and proteins levels in terms of concentrations instead of molecule numbers [72], which is made possible by the PDMP framework. In this view, the cell cycle results in increasing the apparent degradation rates – because of the increase in cell volume followed by division – and thus plays a crucial role for very stable proteins. However, at such a description level, many aspects of possible compensation mechanisms [73] and chromatin dynamics [74] remain to be elucidated. Regarding the latter aspect, our abstract chromatin states were not modeled from real-life data – chromatin composition for instance – but our approach is relevant in that partitioning into dual-type chromatin states as we did is now known as a pervasive feature of all eukaryotic genomes [75–78].

## Conclusions

Protein and mRNA measurements in individual cells have revealed the importance of stochasticity in gene expression, which may potentially affect many aspects of gene regulation within cells. The traditional paradigm of gene network dynamics consisting in a deterministic structure plus an external noise – historically based on population-averaged data – should therefore be questioned, as such a noise appears to be itself part of the network structure and far from a small perturbation.

By modelling gene networks using piecewise-deterministic Markov processes, which are a simple way to introduce the minimum amount of mechanistic, non-diffusive stochasticity (corresponding to low molecule numbers), we derived a likelihood-based statistical model with interpretable parameters that successfully describes single-cell expression data. Our first results show that oriented interactions can indeed be inferred using such a method. Hence, this type of approach may take gene network inference to the next level by optimally exploiting single-cell data and improving the physical interpretability of inferred networks.

## Additional file

Additional file 1
**Additional file 1.** Supplementary information. This document contains details of the theoretical derivations and all the parameter values used in the examples. (PDF 362 kb)
