# Supplementary material for: Inferring gene regulatory networks from single-cell data: a mechanistic approach
Source: BMC Syst Biol. 2017 Nov 21;11:105. doi: 10.1186/s12918-017-0487-0 (PMC5697158; doi:10.1186/s12918-017-0487-0)
Supplement: Additional file 1 — Supplementary information. This document contains details of the theoretical derivations and all the parameter values used in the examples. (PDF 362 kb) [file 12918_2017_487_MOESM1_ESM.pdf]

Inferring gene regulatory networks from single-cell data: a mechanistic approach
– Supplementary information –

Ulysse Herbach[1,2,3*], Arnaud Bonnaffoux[1,2,4], Thibault Espinasse[3], Olivier Gandrillon[1,2]

[1] Univ Lyon, ENS de Lyon, Univ Claude Bernard, CNRS UMR 5239, INSERM U1210, Laboratory of Biology and Modelling of the Cell, 46 allée d'Italie Site Jacques Monod, F-69007 Lyon, France
[2] Inria Team Dracula, Inria Center Grenoble Rhône-Alpes, France
[3] Université de Lyon, Université Lyon 1, CNRS UMR 5208, Institut Camille Jordan 43 blvd du 11 novembre 1918, F-69622 Villeurbanne-Cedex, France
[4] The CoSMo company, 5 passage du Vercors, 69007 Lyon, France

Contents

1 First simplifications **2**
1.1 Normalizing the PDMP network model . . . 2
1.2 Separating mRNA and protein timescales . . . 3

2 Hartree approximtaion **3**
2.1 Hartree approximation for the PDMP model . . . 3
2.2 Solving the reduced problem . . . 4
2.3 Protein marginal distribution . . . 5

3 Explicit interactions **5**
3.1 Simple biochemical model . . . 5
3.2 Stationary distribution . . . 7
3.3 Higher order interactions . . . 7
3.4 The case of auto-activation . . . 8
3.5 Parameterization for inference . . . 8
3.6 Explicit distribution for an auto-activation model . . . 8

4 EM algorithm for network inference **9**
4.1 EM algorithm for MAP estimation . . . 9
4.2 Custom prior on the interactions . . . 10
4.3 The algorithm in practice . . . 11
4.4 Proximal gradient method . . . 12

5 Dealing with real data **14**
5.1 Spreading zeros . . . 14
5.2 Estimating the basal parameters . . . 14

6 Parameter values **15**
6.1 Models . . . 15
6.2 Results . . . 16

* ulysse.herbach@ens-lyon.fr

1 First simplifications

1.1 Normalizing the PDMP network model

In this section we detail the normalization of our network model. Recall that the original model is defined by

$$\begin{cases} E_i(t) : 0 \xrightarrow{k_{\mathrm{on},i}} 1, \quad 1 \xrightarrow{k_{\mathrm{off},i}} 0 \\ M_i{}'(t) = s_{0,i} E_i(t) - d_{0,i} M_i(t) \\ P_i{}'(t) = s_{1,i} M_i(t) - d_{1,i} P_i(t) \end{cases} \tag{1}$$

where $k_{\mathrm{on},i} = k_{\mathrm{on},i}(P_1, \dots, P_n)$ and $k_{\mathrm{off},i} = k_{\mathrm{off},i}(P_1, \dots, P_n)$. First we observe that, given an initial condition

$$(E_1^0, \dots, E_n^0) \in \{0,1\}^n, \quad (M_1^0, \dots, M_n^0) \in \prod_{i=1}^n \left[0, \frac{s_{0,i}}{d_{0,i}}\right], \quad (P_1^0, \dots, P_n^0) \in \prod_{i=1}^n \left[0, \frac{s_{0,i}s_{1,i}}{d_{0,i}d_{1,i}}\right],$$

the system stays in this set for all $t > 0$, and we introduce the dimensionless variables:

$$\overline{M}_i = \frac{d_{0,i}}{s_{0,i}} M_i \in [0,1] \quad \text{and} \quad \overline{P}_i = \frac{d_{0,i}d_{1,i}}{s_{0,i}s_{1,i}} P_i \in [0,1].$$

Then, since $s_{0,i}$, $s_{1,i}$, $d_{0,i}$ and $d_{1,i}$ are constants, we get

$$\overline{M}_i{}'(t) = \frac{d_{0,i}}{s_{0,i}} M_i{}'(t) = d_{0,i}\left(E_i(t) - \frac{d_{0,i}}{s_{0,i}} M_i(t)\right) = d_{0,i}\left(E_i(t) - \overline{M}_i(t)\right)$$

and

$$\overline{P}_i{}'(t) = \frac{d_{0,i}d_{1,i}}{s_{0,i}s_{1,i}} P_i{}'(t) = d_{1,i}\left(\frac{d_{0,i}}{s_{0,i}} M_i(t) - \frac{d_{0,i}d_{1,i}}{s_{0,i}s_{1,i}} P_i(t)\right) = d_{1,i}\left(\overline{M}_i(t) - \overline{P}_i(t)\right).$$

As a result, we obtain the normalized model:

$$\begin{cases} E_i(t) : 0 \xrightarrow{\overline{k_{\mathrm{on},i}}} 1, \quad 1 \xrightarrow{\overline{k_{\mathrm{off},i}}} 0 \\ \overline{M}_i{}'(t) = d_{0,i}\left(E_i(t) - \overline{M}_i(t)\right) \\ \overline{P}_i{}'(t) = d_{1,i}\left(\overline{M}_i(t) - \overline{P}_i(t)\right) \end{cases} \tag{2}$$

where the rescaled interaction function $\overline{k_{\mathrm{on},i}}$ is defined by

$$\overline{k_{\mathrm{on},i}}(\overline{P}_1, \dots, \overline{P}_n) = k_{\mathrm{on},i}\left(\frac{s_{0,1}s_{1,1}}{d_{0,1}d_{1,1}}\overline{P}_1, \dots, \frac{s_{0,n}s_{1,n}}{d_{0,n}d_{1,n}}\overline{P}_n\right)$$

and $\overline{k_{\mathrm{off},i}}$ is defined analogously. It is straightforward to see that, given a path

$$(E_i(t), \overline{M}_i(t), \overline{P}_i(t))_i$$

of the normalized model (2), the corresponding path of the original model (1) is

$$\left(E_i(t), \frac{s_{0,1}}{d_{0,1}}\overline{M}_i(t), \frac{s_{0,1}s_{1,1}}{d_{0,1}d_{1,1}}\overline{P}_i(t)\right)_i.$$

In this sense, both models are equivalent: in the main text and in the next sections, we always consider model (2) but forget the “bars” to keep the notations simple.

1.2 Separating mRNA and protein timescales

Here we justify the reduced network model involving only promoters and proteins, which is valid when $d_{1,i} \ll d_{0,i}$ for all gene i. A full proof is beyond the scope of this article but we provide a heuristic explanation. We temporarily drop the i index for simplicity. Let $t_1 \geqslant t_0 \geqslant 0$ and $E \in \{0,1\}$, and suppose $E(t) = E$ for all $t \in [t_0, t_1]$. Moreover, let $M_0 = M(t_0) \in [0,1]$ and $P_0 = P(t_0) \in [0,1]$. If $d_1 < d_0$, the solution of the linear ODE system

$$\begin{cases} M' = d_0(E - M) \\ P' = d_1(M - P) \end{cases}$$

is given for $t \in [t_0, t_1]$ by

$$\begin{cases} M(t) = E + (M_0 - E)e^{-d_0(t-t_0)} \\ P(t) = E + (P_0 - E)e^{-d_1(t-t_0)} + \dfrac{d_1}{d_0 - d_1}(M_0 - E)\left(e^{-d_1(t-t_0)} - e^{-d_0(t-t_0)}\right) \end{cases}$$

Hence, if $d_1 \ll d_0$, we have

$$P(t) \approx E + (P_0 - E)e^{-d_1(t-t_0)}$$

using the fact that $|M_0 - E| \leqslant 1$ and $|e^{-d_1(t-t_0)} - e^{-d_0(t-t_0)}| \leqslant 1$, and thus $P(t)$ approximates the solution of the differential equation $P' = d_1\,(E - P)$.

2 Hartree approximtaion

2.1 Hartree approximation for the PDMP model

Before deriving the approximation, we introduce some notation. Let n be the number of genes in the network, $\mathcal{E} = \{0,1\}^n$ and $\Omega = (0,1)^n$. At time t, promoter and protein configurations are denoted by $E_t = (e_1, \dots, e_n) = e \in \mathcal{E}$ and $P_t = (y_1, \dots, y_n) = y \in \Omega$, respectively. The distribution of (E_t, P_t) then evolves along time according to its Kolmogorov forward (aka master) equation, which is a linear partial differential equation (PDE) system in our case. This system is high dimensional ($|\mathcal{E}| = 2^n$, the number of possible promoter configurations) but the associated linear operator contains lots of zeros. Using the tensor product notation $\otimes$, one can write down the equation in a compact form:

$$\frac{\partial u}{\partial t} + \sum_{i=1}^{n} \frac{\partial\,(F_i u)}{\partial y_i} = \sum_{i=1}^{n} K_i u \tag{3}$$

where $u(t,y) = (u_e(t,y))_{e\in\mathcal{E}} \in \mathbb{R}^{2^n} \simeq (\mathbb{R}^2)^{\otimes n}$ represents the probability density function (pdf) of (E_t, P_t), and matrices $F_i(y_i)$, $K_i(y) \in \mathcal{M}_{2^n}(\mathbb{R}) \simeq \mathcal{M}_2(\mathbb{R})^{\otimes n}$ are defined by

$$F_i(y_i) = I_2 \otimes \cdots \otimes \underbrace{F^{(i)}(y_i)}_{i} \otimes \cdots \otimes I_2, \qquad K_i(y) = I_2 \otimes \cdots \otimes \underbrace{K^{(i)}(y)}_{i} \otimes \cdots \otimes I_2$$

with

$$F^{(i)}(y_i) = \begin{pmatrix} -d_{1,i}y_i & 0 \\ 0 & d_{1,i}(1-y_i) \end{pmatrix} \quad \text{et} \quad K^{(i)}(y) = \begin{pmatrix} -k_{\mathrm{on},i}(y) & k_{\mathrm{off},i}(y) \\ k_{\mathrm{on},i}(y) & -k_{\mathrm{off},i}(y) \end{pmatrix}.$$

The sum in the left side of equation (3) clearly corresponds to a deterministic transport term, while the right side corresponds to the stochastic transitions between promoter configurations.

Furthermore, the PDE system comes with the boundary condition

$$\forall i \in \{1, \dots, n\}, \quad F_i u = 0 \quad \text{on } \partial\Omega \tag{4}$$

and the probability condition

$$u \geqslant 0 \quad \text{and} \quad \forall t \in \mathbb{R}_+, \quad \sum_{e \in \mathcal{E}} \int_{\Omega} u_e(t, y)\, \mathrm{d}y = 1. \tag{5}$$

The self-consistent "Hartree" approximation consists in splitting this 2^n-dimensional problem into n independent 2-dimensional problems by "freezing" the y_j for $j \neq i$ where i is fixed, and then gathering the solutions by taking their tensor product to produce an approximation of the true pdf (see [1] for a heuristic explanation in the discrete protein setting). More precisely, one reduced problem is derived for each gene i from (3)-(4)-(5):

$$\frac{\partial u^i}{\partial t} + \frac{\partial (F^{(i)} u^i)}{\partial y_i} = K^{(i)} u^i \tag{6}$$

where $u^i(t, y) = (u_0^i(t, y), u_1^i(t, y))^\top \in \mathbb{R}_+^2$ satisfies the initial condition $u^i(0, y) = u^{i,0}(y)$, the boundary condition $F^{(i)}(y_i) u^i(y) \to 0$ when $y_i \to 0$ or 1, and the probability condition $\int_0^1 [u_0^i(t, y) + u_1^i(t, y)]\, \mathrm{d}y_i = 1$ for all $t \geqslant 0$ and $y_1, \dots, y_{i-1}, y_{i+1}, \dots, y_n \in (0, 1)$. Therefore, each u^i is a pdf with respect to $(e_i, y_i) \in \{0, 1\} \times (0, 1)$ but not on $\mathcal{E} \times \Omega$. Finally, the Hartree approximation is given by

$$u(t, y) \approx \bigotimes_{i=1}^{n} u^i(t, y) \tag{7}$$

where the equality holds if for all i, $k_{\mathrm{on},i}$ and $k_{\mathrm{off},i}$ only depend on y_i.

2.2 Solving the reduced problem

For the moment, the time-dependent closed-form solution of (6) is unavailable, but the unique stationary solution can be easily obtained if one knows a primitive of

$$\lambda_i : y_i \mapsto \frac{k_{\mathrm{on},i}(y)}{d_{1,i} y_i} - \frac{k_{\mathrm{off},i}(y)}{d_{1,i}(1 - y_i)}$$

which is the nonzero eigenvalue of the matrix $M^{(i)} = K^{(i)} (F^{(i)})^{-1}$. Indeed, letting $v^i = F^{(i)} u^i$, the stationary equation for v^i from (6) becomes

$$\frac{\partial v^i}{\partial y_i} = M^{(i)} v^i$$

and then, crucially using the fact that $M^{(i)}$ has a constant eigenvector $(-1, 1)^\top$ associated with eigenvalue λ_i (the other eigenvalue being 0), one can check that $v^i = e^{\varphi_i}(-1, 1)^\top$ is a solution when $\frac{\partial \varphi_i}{\partial y_i} = \lambda_i$. If one has such a φ_i, the stationary solution of (6) is given by

$$u_0^i(y) = Z_i^{-1} y_i^{-1} \exp(\varphi_i(y)) \quad \text{and} \quad u_1^i(y) = Z_i^{-1} (1 - y_i)^{-1} \exp(\varphi_i(y)) \tag{8}$$

where Z_i is the normalizing constant (which may still depend on y_j for $j \neq i$). Note that the existence of a positive constant α such that $\min(k_{\mathrm{on},i}, k_{\mathrm{off},i}) \geqslant \alpha$ imposes the limit 0 for $\exp(\varphi_i(y))$ when $y_i \to 0$ or 1, and thus the boundary condition is satisfied. We also obtain the promoter probabilities $p_{0,i} = p(e_i = 0) = Z_{0,i}/Z_i$ and $p_{1,i} = p(e_i = 1) = Z_{1,i}/Z_i$ where $Z_{0,i} = \int_0^1 y_i^{-1} \exp(\varphi_i(y))\, \mathrm{d}y_i$, $Z_{1,i} = \int_0^1 (1 - y_i)^{-1} \exp(\varphi_i(y))\, \mathrm{d}y_i$ and $Z_i = Z_{0,i} + Z_{1,i}$.

In particular, when $k_{\text{on},i}$ and $k_{\text{off},i}$ do not depend on y_i (i.e. no self-interaction), we get

$$\varphi_i(y) = \frac{k_{\text{on},i}(y)}{d_{1,i}} \log(y_i) + \frac{k_{\text{off},i}(y)}{d_{1,i}} \log(1 - y_i)$$

which gives the classical solution

$$u_0^i(y) = \frac{b_i}{a_i + b_i} \cdot \frac{y_i^{a_i - 1}(1 - y_i)^{b_i}}{\mathrm{B}(a_i, b_i + 1)} \quad \text{and} \quad u_1^i(y) = \frac{a_i}{a_i + b_i} \cdot \frac{y_i^{a_i}(1 - y_i)^{b_i - 1}}{\mathrm{B}(a_i + 1, b_i)} \tag{9}$$

with $a_i = k_{\text{on},i}(y)/d_{1,i}$ and $b_i = k_{\text{off},i}(y)/d_{1,i}$. This form makes clear the promoter probabilities $p_{0,i}$ and $p_{1,i}$ and the conditional distributions of protein y_i given the promoter state $e_i = 0$ or 1, both being Beta distributions. Since the state is usually not observed, one usually considers the marginal pdf of y_i, which is also a Beta:

$$\underline{u}^i(y) = u_0^i(y) + u_1^i(y) = \frac{y_i^{a_i - 1}(1 - y_i)^{b_i - 1}}{\mathrm{B}(a_i, b_i)}. \tag{10}$$

Note that the conditional distribution of mRNA given proteins also has the form (10) since the PDMP equation is the same, although the argument is not the Hartree approximation but rather the more common quasi-steady state assumption.

2.3 Protein marginal distribution

Given the form of the solution (8), it is in fact always straightforward to integrate over promoters, even for the full (stationary) Hartree approximation (7), and we finally obtain

$$\underline{u}(y) = \sum_{e \in \mathcal{E}} u_e(y) \approx \sum_{e \in \mathcal{E}} \left[\bigotimes_{i=1}^{n} u^i(y) \right]_e = \sum_{e \in \mathcal{E}} \prod_{i=1}^{n} \frac{\exp(\varphi_i(y))}{Z_i(y)|e_i - y_i|} = \prod_{i=1}^{n} \frac{\exp(\varphi_i(y))}{Z_i(y) y_i (1 - y_i)} \tag{11}$$

where we recalled the possible dependence of Z_i on some y_j. Hence, when φ_i and Z_i are known functions, one gets a fully explicit approximation of the joint protein distribution.

3 Explicit interactions

Here we derive an explicit form for the interactions between genes, starting from a coarse-grained biochemical model. That is, for a given gene i, we focus on defining functions $k_{\text{on},i}(y_1, \dots, y_n)$ and $k_{\text{off},i}(y_1, \dots, y_n)$ where $y_1, \dots, y_n$ denote the protein quantities. For simplicity, we drop the i index in this section when there is no ambiguity.

3.1 Simple biochemical model

The basic idea is to slightly refine the two-state model of gene expression: in addition to the usual switching reactions (whose rates are k_{on} and k_{off}), we consider a set of reversible transitions between some chromatin states (e.g. describing enhancer regions). Each chromatin state is then associated with a particular rate for the promoter activation reaction. For simplicity, we consider only two cases: a high rate k_1 (the chromatin will be said *permissive*) and a low rate $k_0 \ll k_1$ (the chromatin will be said *non-permissive*). Once active, the promoter can switch off at a rate that is supposed to be independent from chromatin states. Finally, we assume that the chromatin transitions are due to fast interactions with ambiant proteins (binding, hit-and-run, etc.) so that the promoter-switching reactions always see chromatin in its quasi-stationary state.

Effective rates k_{on} and k_{off} can therefore be obtained by averaging over chromatin states: this way, k_{off} is still a constant and k_{on} is now defined by

$$k_{\text{on}} = k_0 p_0 + k_1 p_1$$

where p_0 (resp. p_1) is the probability of the chromatin being non-permissive (resp. permissive).

We now define an explicit model for chromatin dynamics and compute its stationary distribution to derive p_0 and p_1 as functions of $y_1, \ldots, y_n$. We consider 2^n permissive configurations and 2^n non-permissive configurations as follows: for all $I \subset \mathcal{G}$ where $\mathcal{G} = \{1, \ldots, n\}$, species C_I (resp. C_I^*) stands for the chromatin being non-permissive (resp. permissive) and in state I. The underlying physics are the following: the chromatin has two "basal" configurations $C_\emptyset$ (non-permissive) and $C_\emptyset^*$ (permissive), which describe dynamics when no protein is present, according to the reactions

$$C_\emptyset \xrightarrow{\alpha} C_\emptyset^*, \quad C_\emptyset^* \xrightarrow{\beta} C_\emptyset.$$

Then, each protein P_j is able to modify the chromatin state through a "hit-and-run" reaction, which is kept in memory by encoding the index j in the list I, giving the state C_I or C_I^*. Eventually, this memory can be lost by "emptying" I step by step (going back to the basal configuration). That is, for all $I \subset \mathcal{G}$ and $j \in \mathcal{G} \setminus I$, we consider the reactions

$$C_I^* + P_j \xrightarrow{a_j} C_{I\cup j}^* + P_j, \quad C_{I\cup j}^* \xrightarrow{b_j} C_I^*,$$

$$C_I + P_j \xrightarrow{c_j} C_{I\cup j} + P_j, \quad C_{I\cup j} \xrightarrow{d_j} C_I.$$

The system then evolves with $[C_I], [C_I^*] \in \{0, 1\}$ and $\sum_I [C_I] + [C_I^*] = 1$, so that only one molecule is present at a time: its species therefore entirely describes the state of the system. Mathematically, we obtain a standard jump Markov process with 2^{n+1} states. For example, the case $n = 2$ leads to the scheme of Figure S1, writing $\bar{a}_j = a_j[P_j]$ and $\bar{c}_j = c_j[P_j]$ for simplicity. The underlying idea is that, depending on a_j, b_j, c_j and d_j, proteins will tend to stabilize the chromatin either in a permissive configuration or in a non-permissive one – providing notions of *activation* and *inhibition*. The basal reactions with rates α and β sum up what we do not observe (i.e. what is likely to happen for the chromatin when none of the P_j are present).

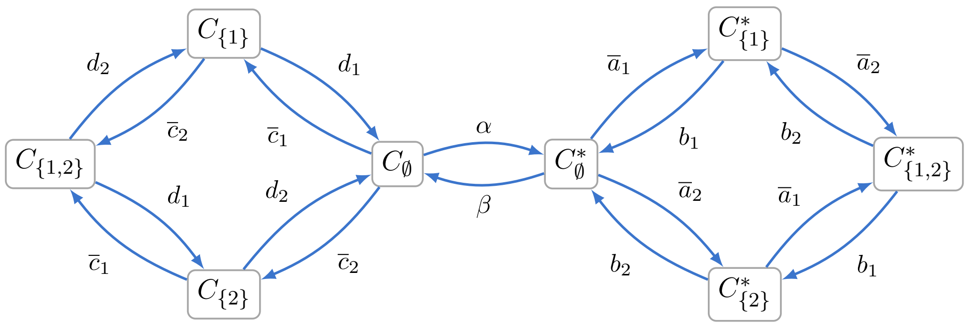

Figure S1: Chromatin states and transitions rates in the case of $n = 2$ proteins.

3.2 Stationary distribution

Letting $\mathcal{S} = \{0,1\}^{n+1}$, each state can be coded by a vector $s = (s_0, s_1, \dots, s_n) \in \mathcal{S}$ where $s_0 = 1$ if the chromatin is permissive and 0 otherwise, and for $j \geqslant 1$, $s_j = 1$ if it has been modified by protein P_j and 0 otherwise. If all rates are positive, the system has a unique stationary distribution π which can be exactly computed from the master equation. More precisely, the probability π_s of the chromatin being in state $s \in \mathcal{S}$ is given by

$$\pi_s = \begin{cases} Z^{-1}\alpha \prod_{j=1}^{n}(\lambda_j[P_j]s_j + 1 - s_j) & \text{if } s_0 = 1 \\ Z^{-1}\beta \prod_{j=1}^{n}(\mu_j[P_j]s_j + 1 - s_j) & \text{if } s_0 = 0 \end{cases}$$

where $\lambda_j = a_j/b_j$, $\mu_j = c_j/d_j$ and Z is a normalizing constant. Now going back to our initial intention of computing k_{on}, we are only interested in the probability for the chromatin to be permissive,

$$p_1 = \sum_{s_1,\dots,s_n} \pi_{(1,s_1,\dots,s_n)} = Z^{-1}\alpha \sum_{s_1,\dots,s_n} \prod_{j=1}^{n} (\lambda_j[P_j]s_j + 1 - s_j),$$

and the probability for the chromatin to be non-permissive,

$$p_0 = \sum_{s_1,\dots,s_n} \pi_{(0,s_1,\dots,s_n)} = Z^{-1}\beta \sum_{s_1,\dots,s_n} \prod_{j=1}^{n} (\mu_j[P_j]s_j + 1 - s_j).$$

Observing that each product term only depends on one s_j, these formulas collapse to

$$p_1 = Z^{-1}\alpha \prod_{j=1}^{n} (\lambda_j[P_j] + 1), \quad p_0 = Z^{-1}\beta \prod_{j=1}^{n} (\mu_j[P_j] + 1)$$

and the distribution condition $p_0 + p_1 = 1$ gives $Z = \alpha \prod_{j=1}^{n}(\lambda_j[P_j]+1) + \beta \prod_{j=1}^{n}(\mu_j[P_j]+1)$. We finally get

$$k_{\text{on}} = \frac{k_0\beta \prod_{j=1}^{n}(\mu_j[P_j]+1) + k_1\alpha \prod_{j=1}^{n}(\lambda_j[P_j]+1)}{\beta \prod_{j=1}^{n}(\mu_j[P_j]+1) + \alpha \prod_{j=1}^{n}(\lambda_j[P_j]+1)}. \tag{12}$$

From this formula, it is straightforward to see that k_{on} will actually depend on a protein P_j only if $\lambda_j \neq \mu_j$, that is, when reactions involving P_j have unbalanced speeds and tend to favor either permissive configurations ($\lambda_j > \mu_j$) or non-permissive configurations ($\lambda_j < \mu_j$).

3.3 Higher order interactions

So far we only considered that the P_j were interacting as monomers. If they in fact interact after forming dimers or other complexes, and if such complex-forming reactions are even faster than chromatin dynamics, one can take this into account by simply replacing $[P_j]$ in equation (12) with a function of $[P_j]$ corresponding to the quasi-stationary concentration of the complex. This approximation seems to be relevant to capture the overall dependence of k_{on} on the proteins, the main point being to use a continuous description (e.g. rate equations) for proteins, which are abundant, while keeping a discrete (stochastic) description for chromatin. We chose to replace $[P_j]$ with $[P_j]^{m_j}$ where $m_j > 0$, which gives our model a general Hill-type form. Note that $m_j = 2$ (resp. $m_j = 3$) may represent a correct approximation for P_j interacting as a dimer (resp. a trimer) but in general m_j does not necessarily have to be an integer.

3.4 The case of auto-activation

A this stage, it is possible to implement self-interaction for gene i by taking $\lambda_i \neq \mu_i$ in (12) but this leads to obvious identifiability issues: in stationary state, one cannot really distinguish between auto-activation, auto-inhibition and basal level. To cope with these, we restrict ourselves to auto-activation by setting $c_i = d_i = 0$ and keeping only the relevant chromatin states (C_I^* for all I, and C_I for I such that $i \notin I$). The system still has a unique stationary distribution and the formula for k_{on} corresponds to the case $\mu_i = 0$ in (12). Then, starting from the fact that auto-activation is only relevant when the basal level is small enough (for a bistable behaviour to be possible), we take the limit $\alpha \ll 1$ while keeping $\alpha\lambda_i$ fixed: the formula becomes

$$k_{\text{on}} = \frac{k_0\beta\prod_{j\neq i}(\mu_j[P_j]^{m_j}+1) + k_1\alpha\lambda_i[P_i]^{m_i}\prod_{j\neq i}(\lambda_j[P_j]^{m_j}+1)}{\beta\prod_{j\neq i}(\mu_j[P_j]^{m_j}+1) + \alpha\lambda_i[P_i]^{m_i}\prod_{j\neq i}(\lambda_j[P_j]^{m_j}+1)} \tag{13}$$

where $m_i > 0$ if gene i activates itself and $m_i = 0$ otherwise.

3.5 Parameterization for inference

Parameters of equation (13) are still clearly not identifiable: in order to get a more minimal form, we introduce the following parameterization: $s_j = {\mu_j}^{-1/m_j}$, $\theta_j = \log(\lambda_j/\mu_j)$ for all $j \neq i$, and $s_i = (\beta/\alpha)^{1/m_i}$, $\theta_i = \log(\lambda_i)$. After simplifying (13), we obtain

$$k_{\text{on}} = \frac{k_0 + k_1\Phi([P_i]/s_i)^{m_i}}{1+\Phi([P_i]/s_i)^{m_i}}$$

where

$$\Phi = \exp(\theta_i)\prod_{j\neq i}\frac{1+\exp(\theta_j)([P_j]/s_j)^{m_j}}{1+([P_j]/s_j)^{m_j}}.$$

The new parameters have an intuitive meaning: s_j can be seen as a threshold for the influence by protein j, and θ_j characterizes this influence via its sign and absolute value ($\theta_j = 0$ implying that k_{on} does not depend on protein j), with the exception that s_i and θ_i aggregate a basal behaviour and an auto-activation strength.

Finally, we recall the notation $y_j = [P_j]$ and reintroduce the index i of the gene of interest and add it to each parameter. Hence, for every gene i, the function $k_{\text{on},i}$ is defined by:

$$k_{\text{on},i}(y) = \frac{k_{0,i} + k_{1,i}\Phi_i(y)(y_i/s_{i,i})^{m_{i,i}}}{1+\Phi_i(y)(y_i/s_{i,i})^{m_{i,i}}} \tag{14}$$

with

$$\Phi_i(y) = \exp(\theta_{i,i})\prod_{j\neq i}\frac{1+\exp(\theta_{i,j})(y_j/s_{i,j})^{m_{i,j}}}{1+(y_j/s_{i,j})^{m_{i,j}}}. \tag{15}$$

In our statistical framework, we assume that parameters $k_{0,i}$, $k_{1,i}$, $m_{i,j}$ and $s_{i,j}$ are known and we focus on inferring the matrix $\theta = (\theta_{i,j}) \in \mathcal{M}_n(\mathbb{R})$, which is similar to the interaction matrix in usual gene network inference methods.

3.6 Explicit distribution for an auto-activation model

Here we derive the stationary distribution for a self-activating gene. For simplicity, we drop the i index. In this model, k_{off} is constant and we assume that there are some constants $\Phi \geqslant 0$, $m \geqslant 0$, $s > 0$ and $k_1 \gg k_0 > 0$ such that k_{on} has the form

$$k_{\text{on}}(y) = \frac{k_0 + k_1\Phi(y/s)^m}{1+\Phi(y/s)^m}$$

so the stationary distribution can directly be used in the Hartree approximation of the network model (14), recalling that Φ has to be independent of the gene's own protein but can depend on others. Letting $c = (k_1 - k_0)/(md_1) > 0$, we are in the case of the explicit solution (8) with

$$\varphi(y) = c \log\left(y^{\frac{k_0}{d_1 c}} + \frac{\Phi}{s^m} y^{\frac{k_1}{d_1 c}}\right) + \frac{k_{\text{off}}}{d_1} \log(1-y)$$

so the protein distribution is

$$\underline{u}(y) = Z^{-1} y^{-1} \left(y^{\frac{k_0}{d_1 c}} + \frac{\Phi}{s^m} y^{\frac{k_1}{d_1 c}}\right)^c (1-y)^{\frac{k_{\text{off}}}{d_1}-1}. \tag{16}$$

To get a fully explicit result, i.e. to compute Z, we shall assume that c is a positive integer. If it is not, one can get a satisfying approximation by taking $c = \lceil (k_1 - k_0)/(md_1) \rceil$. Then, expanding (16) using the binomial theorem, we obtain

$$Z = \sum_{r=0}^{c} \binom{c}{r} \mathrm{B}(a_r, b)(\Phi/s^m)^r$$

where $a_r = ((c-r)k_0 + rk_1)/(d_1 c)$ and $b = k_{\text{off}}/d_1$, and a probabilistic representation of $\underline{u}$ in terms of a mixture of Beta distributions:

$$\underline{u}(y) = \sum_{r=0}^{c} p_r f_r(y) \tag{17}$$

where $f_r(y) = y^{a_r - 1}(1-y)^{b-1}/\mathrm{B}(a_r, b)$ and $p_r = \binom{c}{r}\mathrm{B}(a_r, b)(\Phi/s^m)^r/Z$.

The dissociation constant s is clearly redundant with the input Φ. We fix the particular value

$$s = \left(\frac{\mathrm{B}(a_c, b)}{\mathrm{B}(a_0, b)}\right)^{\frac{1}{mc}} = \left(\frac{\mathrm{B}(k_1/d_1, k_{\text{off}}/d_1)}{\mathrm{B}(k_0/d_1, k_{\text{off}}/d_1)}\right)^{\frac{d_1}{k_1 - k_0}} \tag{18}$$

for which the arbitrary neutral case $\Phi = 1$ is "symmetric", i.e. $p_0 = p_c$. Note that s actually only depends on the fundamental parameters k_0, k_1, k_{off} and d_1 (and not on c nor m). Figure S2 shows some examples of the resulting distribution, which can be bimodal or not, depending on the value of c (or equivalently, m) when all other parameters are fixed.

4 EM algorithm for network inference

4.1 EM algorithm for MAP estimation

Here we briefly recall the formulation of the Expectation-Maximization (EM) algorithm for *maximum a posteriori* (MAP) estimation. Consider the probabilistic hierarchical model defined by the distribution of proteins $p(y|\theta)$, the distribution of mRNA given proteins $p(x|y,\theta)$, and a prior distribution $p(\theta)$ on the parameters. Assuming we only observe x, we want to infer θ by MAP estimation, that is, find a mode – hopefully the highest – of the posterior distribution $p(\theta \,|\, x)$, which satisfies by Baye's rule:

$$p(\theta \,|\, x) = \int p(\theta, y \,|\, x) \,\mathrm{d}y \quad \text{where} \quad p(\theta, y \,|\, x) = p(y \,|\, \theta) p(x \,|\, y, \theta) \frac{p(\theta)}{p(x)}.$$

As $p(\theta \,|\, x)$ has a too complex expression to be efficiently maximized, the EM algorithm rather uses $\ell_\theta(x,y) = \log(p(\theta, y \,|\, x))$ by iteratively computing $\theta^{t+1} = \arg\max_\theta \{Q(\theta, \theta^t)\}$ given θ^t, where

$$\theta \mapsto Q(\theta, \theta^t) = \int \ell_\theta(x,y) p(y \,|\, x, \theta^t) \,\mathrm{d}y. \tag{19}$$

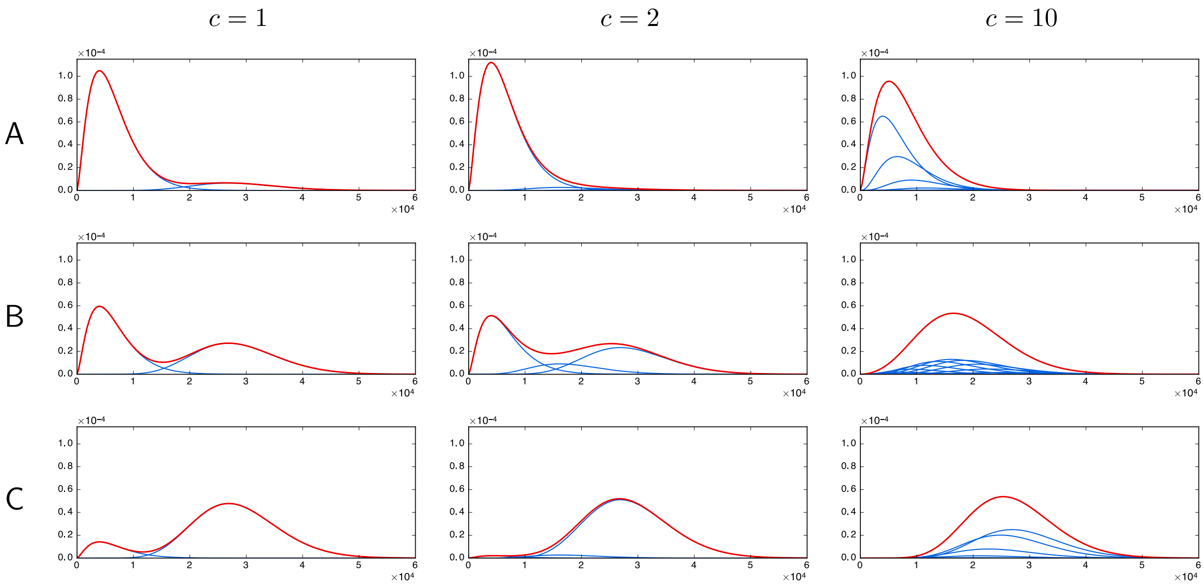

Figure S2: Protein stationary distributions (red curves) from the auto-activation model for different values of the input Φ, with s set as in (18). The blues curves indicate the underlying weighted Beta distributions in each mixture. (A) $\Phi = \exp(-2)$, (B) $\Phi = \exp(0) = 1$, (C) $\Phi = \exp(2)$. The distribution tends to be strongly bimodal for small c values, while large values make the distribution close to the unimodal no-feedback case (constant k_{on}). Parameters are $k_0 = 0.25$, $k_1 = 1.25$, $k_{\text{off}} = 7.5$, $d_1 = 0.1$ and, only used for scaling, $s_1 = 10$, $d_0 = 0.5$, $s_0 = 10^3$.

A well-known result states that at each step we in fact maximize a lower bound of $p(\theta \mid x)$, which is the key point of the algorithm and makes it a particular case of "variational method" (see [2] for example). Now, since $p(x)$ (resp. $p(\theta)$) does not depend on θ (resp. y), it turns out that

$$\arg\max_{\theta}\{Q(\theta, \theta^t)\} = \arg\max_{\theta}\{\overline{Q}(\theta, \theta^t) - g(\theta)\}$$

where $g(\theta) = -\log(p(\theta))$ and $\overline{Q}(\theta, \theta^t) = \int [\log p(y \mid \theta) + \log p(x \mid y, \theta)] p(y \mid x, \theta^t)\, \mathrm{d}y$ is the more standard quantity that appears in the "frequentist" EM algorithm for maximum likelihood estimation. Hence, considering a prior on θ simply results in adding a penalization term $g(\theta)$ during the M step in the algorithm.

For example, if we assume that $\theta_{i,j}$ for $i \neq j$ are independent and follow Laplace distributions, i.e. $p(\theta) = \prod_{i \neq j} \frac{\lambda}{2} \exp(-\lambda |\theta_{i,j}|)$, then $g(\theta) = \lambda \sum_{i \neq j} |\theta_{i,j}| \; + \; C$ where $C = n(n-1)\log(2/\lambda)$. Since C does not depend on θ, this is equivalent to the standard L^1 (lasso) penalization, which is well known to enforce the sparsity of the network.

4.2 Custom prior on the interactions

Here we consider a custom prior to deal with oriented interactions. Indeed, for every pair of nodes $\{i, j\}$ there are two possible interactions with respective parameters $\theta_{i,j}$ and $\theta_{j,i}$, but it is likely that only one is actually present in the true network. Hence, we want $\theta_{i,j}$ and $\theta_{j,i}$ to "compete" against each other so that only one is nonzero after MAP estimation, unless there is enough evidence in the data that both interactions are present. To this aim, we define the

following prior:

$$p(\theta) \propto \exp\left(-\lambda \sum_{i\neq j} |\theta_{i,j}| - \lambda\alpha \sum_{i<j} |\theta_{i,j}\theta_{j,i}|\right) \tag{20}$$

with $\lambda, \alpha \geqslant 0$. Thus α can be seen as a competition parameter, the case $\alpha = 0$ leading to the standard lasso penalization parametrized by λ.

4.3 The algorithm in practice

As visible in (19), the true EM algorithm involves integration against the distribution $p(y \,|\, x, \theta)$, which does not allow for direct numerical integration because of the dimension ($y \in \mathbb{R}^n$). To overcome this problem, a first option is Monte Carlo integration – typically by MCMC – leading to a "stochastic EM" algorithm that is slow but accurate if samples are large enough. A faster option consists in approximating $p(y \,|\, x, \theta)$ by its highest mode, i.e. by the Dirac mass $\delta_{\widehat{y}}$ where $\widehat{y} = \arg\max_y \{p(y \,|\, x, \theta)\}$. Then it is worth noticing that since $p(y \,|\, x, \theta) \propto p(y \,|\, \theta) p(x \,|\, y, \theta)$, the whole procedure can be seen as performing a coordinate ascent on the function $(\theta, y) \mapsto p(\theta, y \,|\, x)$. We chose this option for the examples: it is sometimes called "hard" or "classification" EM, since a particular case leads to the well-known k-means clustering algorithm [3]. Unfortunately, theoretical foundations of the true EM algorithm are lost by the hard EM (we do not maximize a lower bound of $p(\theta \,|\, x)$ anymore), but it often gives satisfying results while requiring much less computational time.

In practice, the procedure is the following. Suppose we observe mRNA levels in m independent cells, and let $\mathbf{x}_k \in \mathbb{R}^n$ (resp. $\mathbf{y}_k \in \mathbb{R}^n$) denote the mRNA (resp. protein) levels of cell k. In line with sections 4.1-4.2 and letting $\mathbf{x} = (\mathbf{x}_1, \ldots, \mathbf{x}_m)$ and $\mathbf{y} = (\mathbf{y}_1, \ldots, \mathbf{y}_m)$ for simplicity, we define the objective function

$$\mathcal{F}(\mathbf{y}, \theta) = \ell(\mathbf{x}, \mathbf{y}, \theta) - g(\theta) \tag{21}$$

where the complete log-likelihood $\ell(\mathbf{x}, \mathbf{y}, \theta)$ and the penalization $g(\theta)$ are given by

$$\ell(\mathbf{x}, \mathbf{y}, \theta) = \sum_{k=1}^{m} \log(u(\mathbf{y}_k, \theta)) + \log(v(\mathbf{x}_k, \mathbf{y}_k, \theta)) \quad \text{and} \quad g(\theta) = \lambda \sum_{i\neq j} |\theta_{i,j}| + \lambda\alpha \sum_{i<j} |\theta_{i,j}\theta_{j,i}|,$$

with $u(y, \theta) = p(y|\theta)$ and $v(x, y, \theta) = p(x|y, \theta)$.

The algorithm then simply consists in iterating the following two steps until convergence:

$$\mathbf{y}^{t+1} = \arg\max_{\mathbf{y}} \{\mathcal{F}(\mathbf{y}, \theta^t)\} \tag{22}$$

$$\theta^{t+1} = \arg\max_{\theta} \{\mathcal{F}(\mathbf{y}^{t+1}, \theta)\} \tag{23}$$

The "approximate E step" (22) can be performed using a standard gradient method since u and v are smooth functions of y. The "penalized M step" (23) is a non-smooth maximization problem since g is non-smooth, but it can be performed using a proximal gradient method detailed in the next section. The form of $\ell(\mathbf{x}, \mathbf{y}, \theta)$ is such that we just need to compute $\nabla \log u$ and $\nabla \log v$.

The formulas for u and v derived from the normalized model are given in the main text: they can be applied once the data has been normalized, i.e. after dividing each mRNA i level by $s_{0,i}/d_{0,i}$. In the bursty regime, this scale parameter is neither identifiable nor necessary. Indeed, as explained in the main text, the "Beta-like" distributions collapse to "Gamma-like" ones which we provide below, and for which the scale parameter is identifiable.

4.3.1 Likelihood form in the basic case

In the basic case (no self-interaction), we have:

$$\log(u(y,\theta)) = \sum_{i=1}^{n}(a_i(y,\theta)-1)\log(y_i) - b_i(y,\theta)y_i + a_i(y,\theta)\log(b_i(y,\theta)) - \log\Gamma(a_i(y,\theta))$$

and

$$\log(v(x,y,\theta)) = \sum_{i=1}^{n}(\widetilde{a}_i(y,\theta)-1)\log(x_i) - \widetilde{b}_i(y,\theta)x_i + \widetilde{a}_i(y,\theta)\log(\widetilde{b}_i(y,\theta)) - \log\Gamma(\widetilde{a}_i(y,\theta))$$

where $a_i = k_{\mathrm{on},i}/d_{1,i}$, $b_i = (d_{0,i}/s_{1,i}) \times (k_{\mathrm{off},i}/s_{0,i})$, $\widetilde{a}_i = k_{\mathrm{on},i}/d_{0,i}$ and $\widetilde{b}_i = k_{\mathrm{off},i}/s_{0,i}$.

4.3.2 Likelihood form in the auto-activation case

In the auto-activation case (section 3.6), the formula for $\log v$ is the same but $\log u$ is given by

$$\log(u(y,\theta)) = \sum_{i=1}^{n}\log\left(\sum_{r=0}^{c_i} w_{i,r}(y,\theta){y_i}^{a_{i,r}-1}e^{-b_i y_i}\right) - \log\left(\sum_{r=0}^{c_i} w_{i,r}(y,\theta)\Gamma(a_{i,r}){b_i}^{-a_{i,r}}\right)$$

where $c_i = \lceil (k_{1,i}-k_{0,i})/(d_{1,i}m_{i,i})\rceil$, $a_{i,r} = ((c_i-r)k_{0,i}+rk_{1,i})/(d_{1,i}c_i)$ and $w_{i,r} = \binom{c_i}{r}(\Phi_i/s_{i,i}^{m_{i,i}})^r$.

4.3.3 Gradients

Explicit computation of the gradients is then straightforward (e.g. with $k_{\mathrm{off},i}$ constant and $k_{\mathrm{on},i}$, Φ_i defined by (14)-(15)) but leads to cumbersome formulas: we implemented them in Scilab and the code is available upon request.

4.4 Proximal gradient method

Here we recall a standard proximal gradient method [4] to solve the M step (23) and provide the proximal operator associated with $g(\theta)$. Note that the method seems to converge in practice, even if g is not convex. It is based on the update

$$\theta^{(k+1)} = \mathrm{prox}_\gamma\left(\theta^{(k)} + \gamma\nabla_\theta \ell(\mathbf{x},\mathbf{y},\theta^{(k)})\right)$$

where $\gamma > 0$ is a step size (learing rate) and prox_γ is the proximal operator associated with $g(\theta)$, defined on $\Theta \simeq \mathbb{R}^{n^2-n}$ by

$$\mathrm{prox}_\gamma(\tau) = \arg\min_{\theta\in\Theta}\left\{g(\theta) + \frac{1}{2\gamma}\sum_{i\neq j}(\theta_{i,j}-\tau_{i,j})^2\right\}.$$

In fact, for any $i,j \in \{1,\dots,n\}$ such that $i \neq j$, one can see that $\theta_{i,j}$ and $\theta_{j,i}$ appear in the minimized quantity as independent of all other θ components. Hence, one just has to compute

$$\mathrm{prox}_\gamma(\tau_1,\tau_2) = \arg\min_{(\theta_1,\theta_2)\in\mathbb{R}^2}\left\{\lambda\left(|\theta_1|+|\theta_2|+\alpha|\theta_1\theta_2|\right) + \frac{1}{2\gamma}\left((\theta_1-\tau_1)^2+(\theta_2-\tau_2)^2\right)\right\}$$

and use it for any $(\tau_1,\tau_2) = (\tau_{i,j},\tau_{j,i}) \in \mathbb{R}^2$ to obtain the corresponding components of $\mathrm{prox}_\gamma(\tau)$. Then, letting $\varepsilon = \lambda\gamma$ and assuming γ small enough such that $\alpha\varepsilon < 1$, we obtain

$$\mathrm{prox}_\gamma(\tau_1,\tau_2) = \frac{1}{1-(\alpha\varepsilon)^2}(h_1,h_2)$$

with 9 cases for the value of (h_1,h_2) depending on (τ_1,τ_2), given by:

1. $\left\{\begin{array}{l}\tau_1 > \varepsilon \\ \tau_1 > \varepsilon(1+\alpha(\tau_2-\varepsilon)) \\ \tau_2 > \varepsilon(1+\alpha(\tau_1-\varepsilon))\end{array}\right. \Rightarrow \left\{\begin{array}{l}h_1 = \tau_1 - \varepsilon(1+\alpha(\tau_2-\varepsilon)) \\ h_2 = \tau_2 - \varepsilon(1+\alpha(\tau_1-\varepsilon))\end{array}\right.$

2. $\left\{\begin{array}{l}\tau_1 > \varepsilon \\ |\tau_2| \leqslant \varepsilon(1+\alpha(\tau_1-\varepsilon))\end{array}\right. \Rightarrow \left\{\begin{array}{l}h_1 = \tau_1 - \varepsilon \\ h_2 = 0\end{array}\right.$

3. $\left\{\begin{array}{l}\tau_1 > \varepsilon \\ \tau_1 > \varepsilon(1+\alpha(-\tau_2-\varepsilon)) \\ \tau_2 < -\varepsilon(1+\alpha(\tau_1-\varepsilon))\end{array}\right. \Rightarrow \left\{\begin{array}{l}h_1 = \tau_1 - \varepsilon(1+\alpha(-\tau_2-\varepsilon)) \\ h_2 = \tau_2 + \varepsilon(1+\alpha(\tau_1-\varepsilon))\end{array}\right.$

4. $\left\{\begin{array}{l}|\tau_1| \leqslant \varepsilon(1+\alpha(-\tau_2-\varepsilon)) \\ \tau_2 < -\varepsilon\end{array}\right. \Rightarrow \left\{\begin{array}{l}h_1 = 0 \\ h_2 = \tau_2 + \varepsilon\end{array}\right.$

5. $\left\{\begin{array}{l}\tau_1 < -\varepsilon \\ \tau_1 < -\varepsilon(1+\alpha(-\tau_2-\varepsilon)) \\ \tau_2 < -\varepsilon(1+\alpha(-\tau_1-\varepsilon))\end{array}\right. \Rightarrow \left\{\begin{array}{l}h_1 = \tau_1 + \varepsilon(1+\alpha(-\tau_2-\varepsilon)) \\ h_2 = \tau_2 + \varepsilon(1+\alpha(-\tau_1-\varepsilon))\end{array}\right.$

6. $\left\{\begin{array}{l}\tau_1 < -\varepsilon \\ |\tau_2| \leqslant \varepsilon(1+\alpha(-\tau_1-\varepsilon))\end{array}\right. \Rightarrow \left\{\begin{array}{l}h_1 = \tau_1 + \varepsilon \\ h_2 = 0\end{array}\right.$

7. $\left\{\begin{array}{l}\tau_1 < -\varepsilon \\ \tau_1 < -\varepsilon(1+\alpha(\tau_2-\varepsilon)) \\ \tau_2 > \varepsilon(1+\alpha(-\tau_1-\varepsilon))\end{array}\right. \Rightarrow \left\{\begin{array}{l}h_1 = \tau_1 + \varepsilon(1+\alpha(\tau_2-\varepsilon)) \\ h_2 = \tau_2 - \varepsilon(1+\alpha(-\tau_1-\varepsilon))\end{array}\right.$

8. $\left\{\begin{array}{l}|\tau_1| \leqslant \varepsilon(1+\alpha(\tau_2-\varepsilon)) \\ \tau_2 > \varepsilon\end{array}\right. \Rightarrow \left\{\begin{array}{l}h_1 = 0 \\ h_2 = \tau_2 - \varepsilon\end{array}\right.$

9. $\left\{\begin{array}{l}|\tau_1| \leqslant \varepsilon \\ |\tau_2| \leqslant \varepsilon\end{array}\right. \Rightarrow \left\{\begin{array}{l}h_1 = 0 \\ h_2 = 0\end{array}\right.$

These 9 cases form a partition of $\mathbb{R}^2$ and are represented in Figure S3. One can check that the case $\alpha = 0$ collapses to the usual proximal operator associated with lasso penalization.

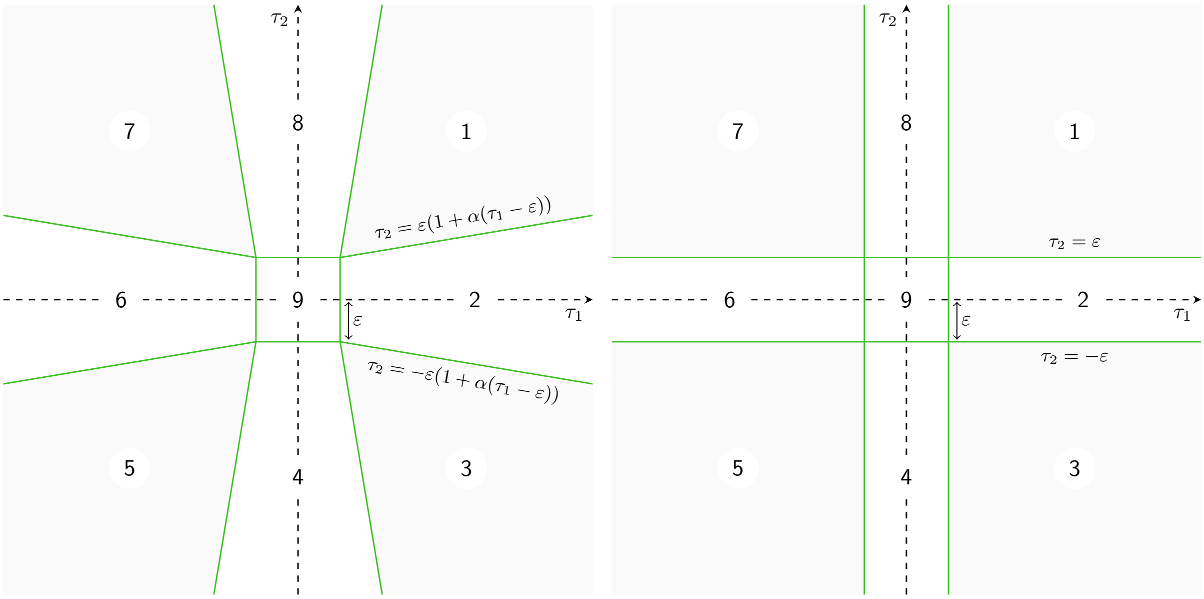

Figure S3: Partition of $\mathbb{R}^2$ associated with the proximal operator, for $\alpha > 0$ (left) and $\alpha = 0$ (right). Gray areas correspond to a usual gradient and white areas correspond to a threshold.

To obtain the results of Fig 8, we used $\lambda = 10$, $\alpha = 5$ and $\gamma = 10^{-4}$. In a broader context, one may use standard cross-validation to derive appropriate values for λ and α.

5 Dealing with real data

In this section, we propose a pre-processing phase which would be required in order to apply our network inference method to real data. The first step ensures that the approximate likelihood is well-defined, while the second step consists in estimating the basal parameters appearing in functions $k_{\text{on},i}$, $k_{\text{off},i}$. Please note that inferring real networks from real data is beyond the scope of this paper and will be the subject of future papers.

5.1 Spreading zeros

The likelihood does not accept exact zeros (cf. section 4.3). This is not a problem with continuous-type data (for instance, based on fluorescence measurements), but it becomes one when dealing with counts (e.g. RNA-Seq). We propose to replace such zeros with relevant positive values. Recall that the PDMP focuses on the promoter and neglects the local molecular noise at the mRNA level. It is therefore natural to consider that, given a value $M > 0$ of mRNA level in the PDMP, the actual number m of molecules in the cell is drawn from the Poisson distribution $\mathcal{P}(M)$. Then, a possible way to replace zeros is to go backwards, i.e. to draw a value M from the PDMP distribution conditioned to $m = 0$. Namely, we propose the following procedure to be applied independently for each gene:

1. Infer a gamma distribution $\gamma(a, b)$ (as a local approximation of the PDMP) from the whole data (possibly at a given time-point) using the standard method of moments;
2. Replace zeros with independent samples from the distribution $\gamma(a, b + 1)$, conditioned to be smaller than the smallest positive value that was measured.

This procedure ensures that zeros are replaced with very small values and that no artificial correlation is introduced. The distribution $\gamma(a, b+1)$ comes from the fact that, if $\mathcal{L}(M) = \gamma(a, b)$ and $\mathcal{L}(m|M) = \mathcal{P}(M)$, then a simple computation gives $\mathcal{L}(M|m = 0) = \gamma(a, b + 1)$.

5.2 Estimating the basal parameters

Here we describe a heuristic method to estimate the model-specific parameters (i.e. everything but the matrix θ) when they cannot be measured through *ad hoc* experiments, in the case of the auto-activation form (14)-(15). Once again we refer to section 4.3. Note that for the mechanistic approach to be relevant, one should know at least the ratio $d_{0,i}/d_{1,i}$, which can be obtained by measuring mRNA and protein half-lives. When even this is unavailable, we propose to use the default value $d_{0,i}/d_{1,i} = 5$ (mean value derived from the literature, cf. main text).

The main idea consists in noticing that, when protein i is described by the auto-activation model (14)-(15) (thus following the distribution (17)), mRNA i in quasi-steady state happens to be well described by the same distribution class as (17), with the same $m_{i,i}$ and other parameters being divided by $d_{0,i}/d_{1,i}$. More precisely, we perform the following steps:

1. Estimate $\widetilde{a}_{0,i} = k_{0,i}/d_{0,i}$, $\widetilde{a}_{1,i} = k_{1,i}/d_{0,i}$, $\widetilde{b}_i = k_{\text{off},i}/s_{0,i}$, $\widetilde{c}_i$ and Φ from the likelihood

$$f(x_i) \propto \sum_{r=0}^{\widetilde{c}_i} \Phi^r {x_i}^{(1-r/\widetilde{c}_i)\widetilde{a}_{0,i}+(r/\widetilde{c}_i)\widetilde{a}_{1,i}-1} e^{-\widetilde{b}_i x_i}.$$

   This can be done for instance using an EM algorithm for each value of $\widetilde{c}_i$ in some range (e.g. $\widetilde{c}_i = 1, 2, \ldots, 10$), and then choosing the "arg max" tuple $(\widetilde{a}_{0,i}, \widetilde{a}_{1,i}, \widetilde{b}_i, \widetilde{c}_i, \Phi)$. Afterwards, $\widetilde{a}_{0,i}$, $\widetilde{a}_{1,i}$, $\widetilde{b}_i$ and $\widetilde{c}_i$ are stored (Φ only serves this step).

2. Consistently with the definition of the model, we set $m_{i,i} = (\widetilde{a}_{1,i} - \widetilde{a}_{0,i})/\widetilde{c}_i$,

$$a_{0,i} = \frac{k_{0,i}}{d_{1,i}} = \frac{d_{0,i}}{d_{1,i}} \cdot \widetilde{a}_{0,i}, \quad a_{1,i} = \frac{k_{1,i}}{d_{1,i}} = \frac{d_{0,i}}{d_{1,i}} \cdot \widetilde{a}_{1,i}, \quad c_i = \frac{k_{1,i} - k_{0,i}}{d_{1,i} m_{i,i}} = \frac{d_{0,i}}{d_{1,i}} \cdot \widetilde{c}_i$$

and we choose $b_i = \frac{d_{0,i}}{d_{1,i}} \cdot \widetilde{b}_i$. Then we define, as an approximation of (18) in the bursty regime,

$$s_{i,i} = \frac{1}{b_i} \left(\frac{\Gamma(a_{1,i})}{\Gamma(a_{0,i})} \right)^{1/(a_{1,i} - a_{0,i})}.$$

Note that such b_i is not the "true" value regarding section 4.3.2, as we would need to know $\frac{d_{1,i}}{s_{1,i}}$ to apply the formula $b_i = \frac{d_{1,i}}{s_{1,i}} \cdot \frac{d_{0,i}}{d_{1,i}} \cdot \widetilde{b}_i$. Fortunately, the network inference does not depend on this scale parameter since the Hill threshold $s_{i,i}$ is proportional to $1/b_i$.

3. Last step consists in extrapolating $m_{i,i}$ and $s_{i,i}$ to the remaining unknown parameters $m_{i,j}$ and $s_{i,j}$ (describing how gene j influences gene i). Since the crucial point is their coherence with respect to the range of protein j, a relevant choice without additional knowledge is, for all $i \neq j$,

$$m_{i,j} = m_{j,j} \quad \text{and} \quad s_{i,j} = s_{j,j}.$$

6 Parameter values

6.1 Models

Table S1: General parameters used in the examples. The $s_{i,j}$ correspond to the normalized model: counterparts in absolute protein numbers are $\overline{s}_{i,j} = s_{i,j} \times (s_0 s_1)/(d_0 d_1) = 2 \times 10^3$ for $i \neq j$ and $\overline{s}_{i,i} = s_{i,i} \times (s_0 s_1)/(d_0 d_1) = 1.9 \times 10^4$.

Parameter	Value	Units
s_0	10^3	mRNA $\cdot$ h^{-1}
s_1	10	protein $\cdot$ h^{-1} $\cdot$ mRNA^{-1}
d_0	0.5	h^{-1}
d_1	0.1	h^{-1}
k_0	0.34	h^{-1}
k_1	2.15	h^{-1}
k_{off}	10	h^{-1}
$m_{i,j}$	2 for $i \neq j$	–
$m_{i,i}$	2 (Fig 5, 6) or 3 (Fig 8)	–
$s_{i,j}$	0.01 for $i \neq j$	proteins (normalized)
$s_{i,i}$	0.095 from eq. (18)	proteins (normalized)

Table S2: Network parameters used in the examples.

Fig 5, 6	$\theta_{1,1}$	$\theta_{1,2}$	$\theta_{2,1}$	$\theta_{2,2}$
	4	−8	−8	4
**Fig 8**	$\theta_{1,1}$	$\theta_{1,2}$	$\theta_{2,1}$	$\theta_{2,2}$
1	0	0	0	0
2	0	0	1	0
3	0	1	0	0
4	−0.1	1	1	−0.1
5	0	0	−1	0
6	0	−1	0	0
7	0	−1	−1	0

6.2 Results

Table S3: Inferred network parameters used to generate Fig 8b. Each row refers to one of the ten datasets generated for testing. Colors indicate whether the parameters represent the correct topology (blue) or another one (orange) regarding the true networks (cf. Table S2).

	Network 1		Network 2		Network 3		Network 4	
	$\theta_{1,2}$	$\theta_{2,1}$	$\theta_{1,2}$	$\theta_{2,1}$	$\theta_{1,2}$	$\theta_{2,1}$	$\theta_{1,2}$	$\theta_{2,1}$
1	0	0	0	0.13	0	0	0.28	0.22
2	0	0	0	0.18	0.12	0	0.25	0.15
3	0	0	0	0.14	0.26	0.10	0.23	0.21
4	0	0	0	0.17	0.19	0	0.20	0.15
5	0.10	0	0	0	0.21	0	0.18	0.23
6	0	0	0	0.20	0.36	0.15	0.23	0.27
7	0	0	0	0.18	0.11	0	0.17	0.16
8	0	0	0	0.11	0.11	0	0.14	0.13
9	0	0	0	0.15	0.19	0	0.29	0.21
10	0	0	0	0	0.12	0	0.24	0.21

	Network 5		Network 6		Network 7	
	$\theta_{1,2}$	$\theta_{2,1}$	$\theta_{1,2}$	$\theta_{2,1}$	$\theta_{1,2}$	$\theta_{2,1}$
1	0	0	−0.14	0	−0.20	−0.24
2	0	−0.12	−0.11	0	−0.34	−0.34
3	0	−0.10	−0.13	0	−0.17	−0.14
4	0	−0.16	−0.15	0	−0.23	−0.20
5	−0.10	−0.21	−0.21	0	−0.17	−0.18
6	0	−0.18	−0.16	0	−0.19	−0.16
7	0	−0.13	−0.29	−0.09	−0.21	−0.21
8	0	−0.09	0	0	−0.30	−0.31
9	0	0	−0.13	0	−0.11	−0.13
10	0	−0.18	−0.21	0	−0.11	−0.17

Table S4: Example of inferred networks in the presence of dropouts (30% of the whole dataset) generated by applying a Poisson noise and then a threshold to the "perfect" data. Such zeros where replaced using the procedure described in section 5.1 before inferring the networks.

	Network 1		Network 2		Network 3		Network 4	
	$\theta_{1,2}$	$\theta_{2,1}$	$\theta_{1,2}$	$\theta_{2,1}$	$\theta_{1,2}$	$\theta_{2,1}$	$\theta_{1,2}$	$\theta_{2,1}$
1	0	0	0	0.27	0.31	0	0.43	0.41
2	0	0.10	0	0.16	0.23	0	0.44	0.26
3	0	0	0	0.31	0.32	0.12	0.36	0.28
4	0	0	0	0.44	0.30	0	0.44	0.23
5	0.10	0	0	0.27	0.39	0.11	0.38	0.51
6	0	0	0	0.55	0.40	0.16	0.42	0.48
7	0.09	0.16	0	0.23	0.19	0.11	0.48	0.34
8	0	0	0	0.21	0.14	0	0.36	0.32
9	0	0	0	0.36	0.35	0.16	0.59	0.43
10	0	0	0	0	0.19	0	0.41	0.34

	Network 5		Network 6		Network 7	
	$\theta_{1,2}$	$\theta_{2,1}$	$\theta_{1,2}$	$\theta_{2,1}$	$\theta_{1,2}$	$\theta_{2,1}$
1	0	0	−0.18	0	−0.22	−0.24
2	0	−0.26	−0.25	0	−0.5	−0.50
3	−0.09	−0.21	−0.28	0	−0.25	−0.21
4	−0.11	−0.35	−0.25	0	−0.26	−0.22
5	−0.30	−0.47	−0.34	0	−0.12	−0.14
6	0	−0.32	−0.26	0	−0.20	−0.18
7	0	−0.22	−0.47	−0.18	−0.30	−0.29
8	0	−0.19	−0.09	0	−0.44	−0.45
9	0	−0.27	−0.22	0	−0.15	−0.18
10	0	−0.38	−0.38	−0.12	−0.11	−0.17

References

[1] A. M. Walczak, M. Sasai, and P. G. Wolynes, "Self-consistent proteomic field theory of stochastic gene switches," *Biophysical Journal*, vol. 88, pp. 828–850, 2005.

[2] M. I. Jordan, Z. Ghahramani, T. S. Jaakkola, and L. K. Saul, "An Introduction to Variational Methods for Graphical Models," *Machine Learning*, vol. 37, no. 2, pp. 183–233, 1999.

[3] G. Celeux and G. Govaert, "A classification EM algorithm for clustering and two stochastic versions," Research Report 1364, INRIA, 1991.

[4] N. Parikh and S. Boyd, "Proximal Algorithms," *Foundations and Trends in Optimization*, vol. 1, no. 3, pp. 123–231, 2013.
